# Alkyl chain functionalised Ir(iii) complexes: synthesis, properties and behaviour as emissive dopants in microemulsions†

**DOI:** 10.1039/d3ra06764e

**Published:** 2024-02-27

**Authors:** Emily C. Stokes, Ibrahim O. Shoetan, Alice M. Gillman, Peter N. Horton, Simon J. Coles, Simon E. Woodbury, Ian A. Fallis, Simon J. A. Pope

**Affiliations:** a School of Chemistry, Cardiff University Main Building Cardiff CF10 3AT UK popesj@cardiff.ac.uk; b Chemistry, UK National Crystallographic Service, Faculty of Natural and Environmental Sciences, University of Southampton Highfield Southampton SO17 1BJ England UK; c National Nuclear Laboratory, Central Laboratory Sellafield, Seascale Cumbria CA20 1PG UK

## Abstract

Six iridium(iii) complexes of the general form [Ir(C^N)_2_(N^N)]X (where C^N = cyclometalating ligand; N^N = disubstituted 2,2′-bipyridine), and incorporating alkyl chains of differing lengths (C8, C10, C12), have been synthesised and characterised. The complexes have been characterised using a variety of methods including spectroscopies (NMR, IR, UV-Vis, luminescence) and analytical techniques (high resolution mass spectrometry, cyclic voltammetry, X-ray diffraction). Two dodecyl-functionalised complexes were studied for their behaviour in aqueous solutions. Although the complexes did not possess sufficient solubility to determine their critical micelle concentrations (CMC) in water, they were amenable for use as emissive dopants in a *N*-methyl C12 substituted imidazolium salt microemulsion carrier system with a CMC = 36.5 mM. The investigation showed that the metal doped microemulsions had increased CMCs of 40.4 and 51.3 mM and luminescent properties characterised by the dopant.

## Abstract 2

Luminescent, alkyl chain functionalised Ir(iii) complexes have been synthesised, characterised and studied in microemulsion environments showing retention of emissive behaviour.

## Graphical abstract

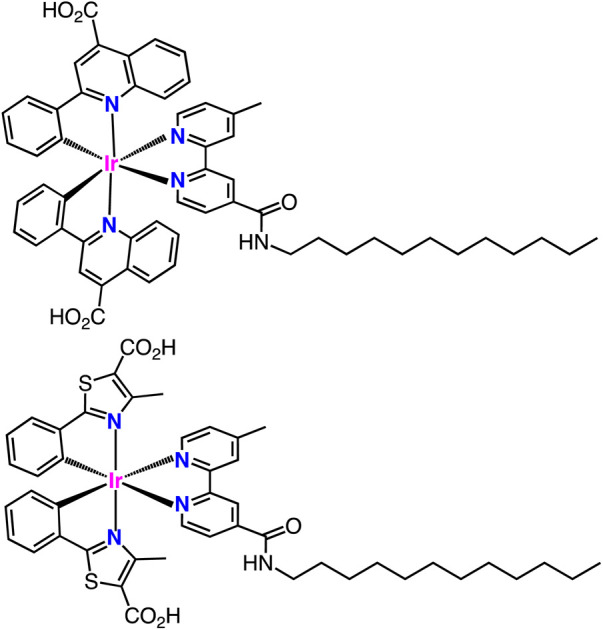

## Introduction

Amphiphilic metal ion coordination complexes that have been investigated in the context of micellar or microemulsion materials^1^ are known as metallosurfactants.^2^ Interest in metallosurfactants has been driven by a number of applications within catalysis, biological and biomedical disciplines. For example, 1,10-phenanthroline-derived Zn(ii) complexes that incorporate long alkyl chains combined with cetyltrimethylammonium bromide (a cationic surfactant) form micellar systems that have been reported as catalysts for the hydrolysis of *p*-nitrophenyl picolinate species.^3^ Similarly, azamacrocyclic Zn(ii) metallosurfactants have been studied for the catalytic hydrolysis of lipophilic esters.^4^ Amphiphilic phosphine ligands have been developed to yield Pd(ii) metallosurfactants which exhibit potential for a variety of catalytic transformations.^5^ Triazacyclododecane ligands which incorporate polymerisable vinylbenzene sidearms can form metallosurfactants with Ni(ii), Cu(i), Cu(ii), and Co(ii).^6^ Cu(ii)-containing surfactants have also been found to be effective catalysts in the hydrolysis of the nerve agent sarin and related phosphates.^7^ Metallosurfactants based upon N-heterocyclic carbene Cu(i) and Fe(ii) complexes have shown promise as emulsion polymerization catalysts.^8^

One of the more commonly studied d-block metals for metallosurfactants is Ru(ii), including the use of analogues of [Ru(bipy)_3_](2Cl) that incorporate long alkyl chains; applications included the formulation of thin films and heterogeneous catalysis.^9,10^ Small angle neutron scattering (SANS) studies revealed a change in micelle morphology as the length of the alkyl chain was increased (*n* = 12, 15, 19).^11^ Thin films of these complexes can be calcined to yield metal-containing particles within highly ordered mesoporous materials for catalysis.^12,13^

The photoactive properties of coordination complexes can also be exploited using metallosurfactants. For example, [Fe(CN)_2_L_2_] (where L is a symmetric or asymmetric 2,2′-bipyridine analogue functionalised with different alkyl chain lengths) complexes have been investigated as potential solvatochromic probes in organised media.^14^ Recent work has shown that amphiphilic luminescent Ir(iii) complexes can be used to form biologically active micelles which possess low dark toxicity and phototherapeutic application.^15^ A PEG-ylated luminescent Ir(iii) complex has also been demonstrated to self-assemble as a micellar material and has been subsequently utilised for selective tumour imaging.^16^

**Fig. 1:**
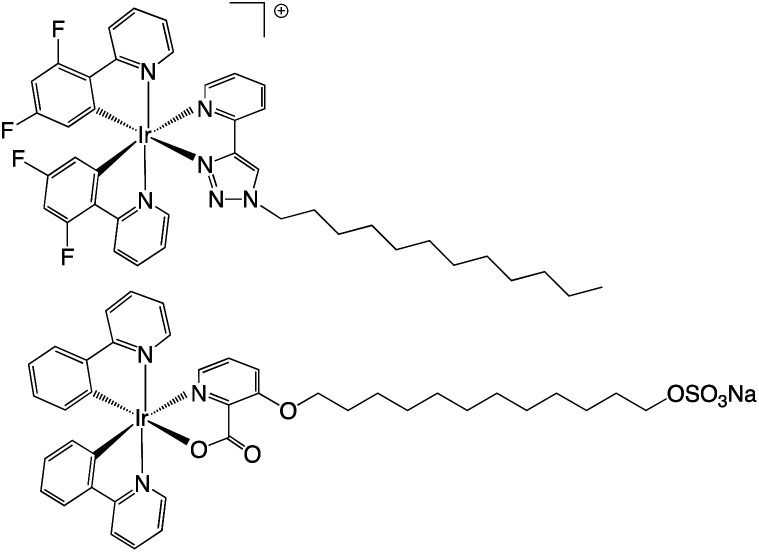
Two examples of amphiphilic Ir(iii) complexes previously reported as metallosurfactants.

The work of de Cola on amphiphilic cyclometalated Ir(iii) complexes is of direct relevance to the current study. The work described different strategies for attaching varying alkyl chain moieties to Ir(iii) complexes resulting in either cationic or anionic amphiphiles with surfactant properties (Fig. 1). Zwitterionic examples of the complexes show aggregation induced enhancement^17^ or tuning of emission properties in the solid state.^18^

The aim of the current work was to develop a series of luminescent, alkyl chain functionalised Ir(iii) complexes which could then be converted into emissive, potentially amphiphilic, species in water. The self-organised features of aqueous surfactant solutions can give rise to unique photophysical properties when one or more components of the surfactant are photoactive.^19^ We were therefore interested to investigate the potential and utility of alkyl chain derived Ir(iii) complexes as dopants of micellar microemulsion systems to produce new luminescent colloidal materials.

## Results and discussion

### Synthesis

**Scheme 1:**
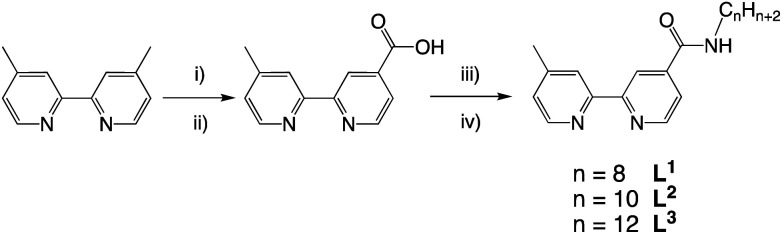
Synthetic route to the ligands, L^1–3^. Reagents and conditions: (i) SeO_2_, dioxane, heat; (ii) Ag_2_O, EtOH; (iii) SOCl_2_, MeCN; (iv) 1-octylamine/decylamine/dodecylamine, DIPEA, MeCN.

The desired properties of the amphiphilic Ir(iii) species were targeted through a combination of ligands to yield heteroleptic luminescent complexes. Firstly, different alkyl chain lengths (octyl, decyl, and dodecyl) were added to a 4,4′-functionalised variant of 2,2′-bipyridine. This was achieved using 4-methyl-2,2′-bipyridine-4′-carboxylic acid^20^ (synthesised from commercially available 4,4′-dimethyl-2,2′-bipyridine) to conveniently yield the amide linked target ligands, L^1–3^ (Scheme 1). We hypothesised that the amide link may subtly assist the hydrophilic character of the complexes.

**Scheme 2:**
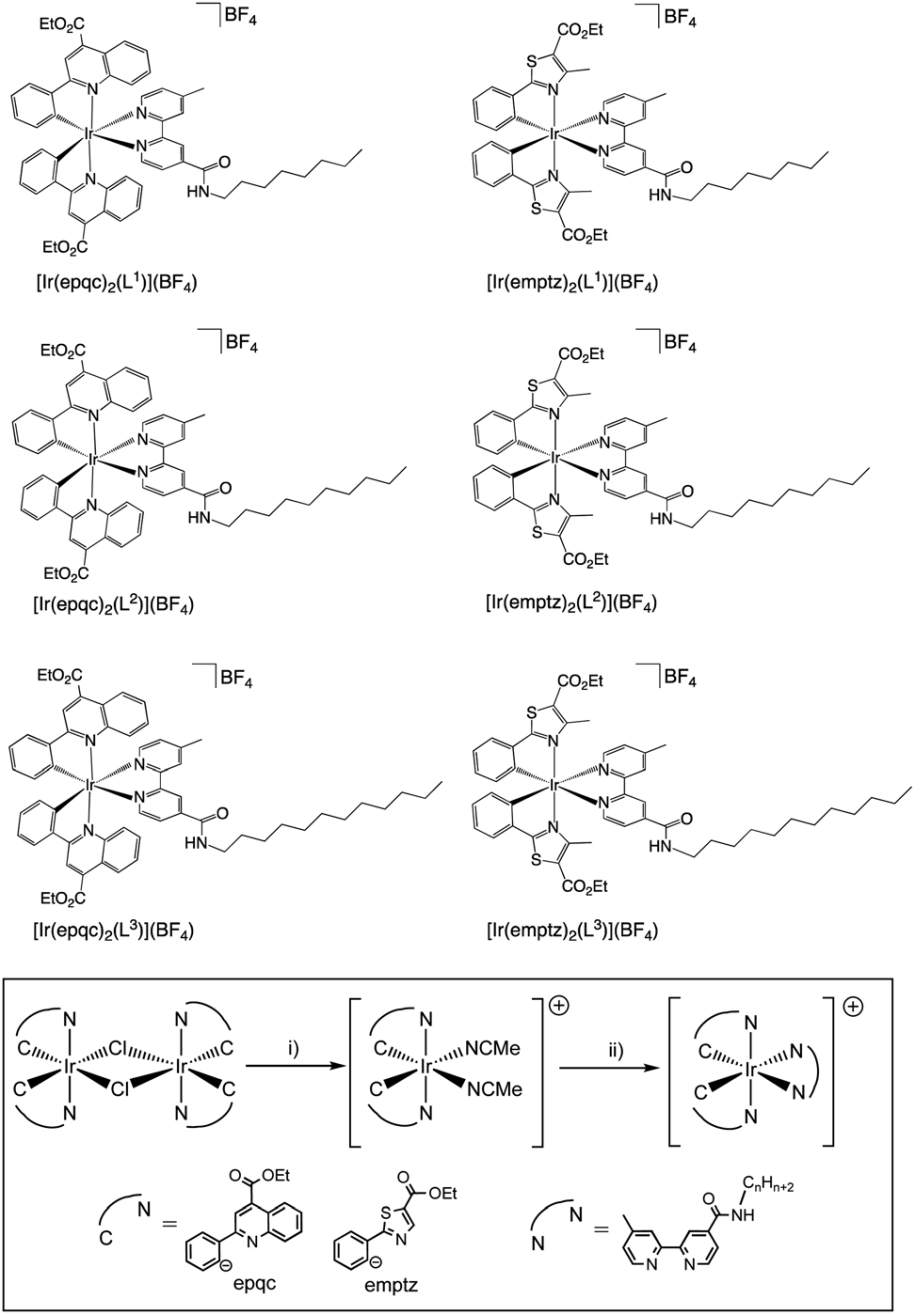
Structures of the ester-functionalised iridium(iii) complexes synthesised in this study. The general route to the complexes shown inset. Reagents and conditions: (i) AgBF_4_, MeCN, heat; (ii) L^1^/L^2^/L^3^, CHCl_3_, heat.

Secondly, two different cyclometalating ligands were selected that incorporate an ester functionality: ethyl-2-phenylquinoline-4-carboxylate (epqcH) and ethyl-4-methylphenylthiazole-5-carboxylate (emptzH). Both ligands therefore enable hydrophilic character to be imparted through the subsequent deprotection of the ester group (see later discussion). In the complex synthesis, two chloro-bridged Ir(iii) dimers [{Ir(C^N)_2_-(μ-Cl)}_2_] (C^N = epqc, emptz) were isolated following a previous method (Scheme 2).^21^ The Ir(iii) dimers were then split using MeCN in the presence of AgBF_4_ to yield the intermediate monometallic complexes *cis*-[Ir(C^N)_2_(MeCN)_2_]BF_4_. The two bis-acetonitrile complexes were then further reacted with the different bipyridine ligands L^1–3^ to give cationic, ester-functionalised bis-cyclometalated complexes [Ir(C^N)_2_(L^1–3^)]BF_4_ (Scheme 2) as bright red to dark brown coloured solids in moderate-to-high yields.

Each of the bipyridine ligands, L^1^–L^3^, were characterised *via* UV-Vis, IR, ^1^H and ^13^C{^1^H} NMR spectroscopies and mass spectrometry (Fig. S1–S8†). ^1^H NMR spectra confirmed the unsymmetrical nature of the disubstituted bipyridine with a series of unique aromatic resonances, and the presence of the amide N*H* group with a broadened singlet around 6.7 ppm. ^13^C{^1^H} NMR spectra revealed the C=O resonance of the amide unit to be *ca.* 165 ppm. IR spectra supported the C=O assignment with a vibration around 1630 cm^−1^ consistent with an amide group.

For the intermediate bis-acetonitrile Ir(iii) complexes, *cis*-[Ir(C^N)_2_(MeCN)_2_]BF_4_, the ^1^H NMR spectra of these species gave key resonances for the coordinated MeCN ligands at 2.43 ppm for *cis*-[Ir(emptz)_2_(MeCN)_2_]BF_4_ and 2.24 ppm for *cis*-[Ir(epqc)_2_(MeCN)_2_]BF_4_. The most upfield resonances of the ^13^C NMR spectra were attributed to the methyl group of the coordinated acetonitrile ligands.^22^ The corresponding IR spectra of these precursors showed peaks at 2311 and 2322 cm^−1^ for *cis*-[Ir(emptz)_2_(MeCN)_2_]BF_4_ and *cis*-[Ir(epqc)_2_(MeCN)_2_]BF_4_ respectively, consistent with *ν*(C≡N) associated with a coordinated nitrile ligand.

For [Ir(emptz)_2_(L)]BF_4_ and [Ir(epqc)_2_(L)]BF_4_, the unsymmetrical nature of the coordinated ancillary ligand (L^1–3^) led to a large number of aromatic resonances in both ^1^H and ^13^C NMR spectra. For the ^1^H NMR spectra, all cases showed that the relative total integration of the alkyl chain protons were consistent with the proposed formulations. For [Ir(emptz)_2_(L^1–3^)]BF_4_ and [Ir(epqc)_2_(L^1–3^)]BF_4_ the spectra showed that the ester moieties of the cyclometalated ligand components were retained with no evidence of hydrolysis or transesterification (with 2-methoxyethanol) as has been occasionally noted using the reaction conditions.^23^ The unsymmetrical nature of the heteroleptic complexes was also exemplified by the observation of three unique carbonyl resonances (between 162 and 185 ppm) in the ^13^C NMR spectra; these are attributed to the esters of the two C^N ligands, and the amide group of the coordinated bipyridine. IR spectra confirmed the loss of the *ν*(C≡N) bands of the bis-acetonitrile precursors, and the presence of different C=O vibrational frequencies associated with the ester and amide groups. All relevant details and data are provided in the Experimental Section and the ESI (Fig. S9–S25†).

### Structural characterisation *via* X-ray crystallography studies

**Fig. 2:**
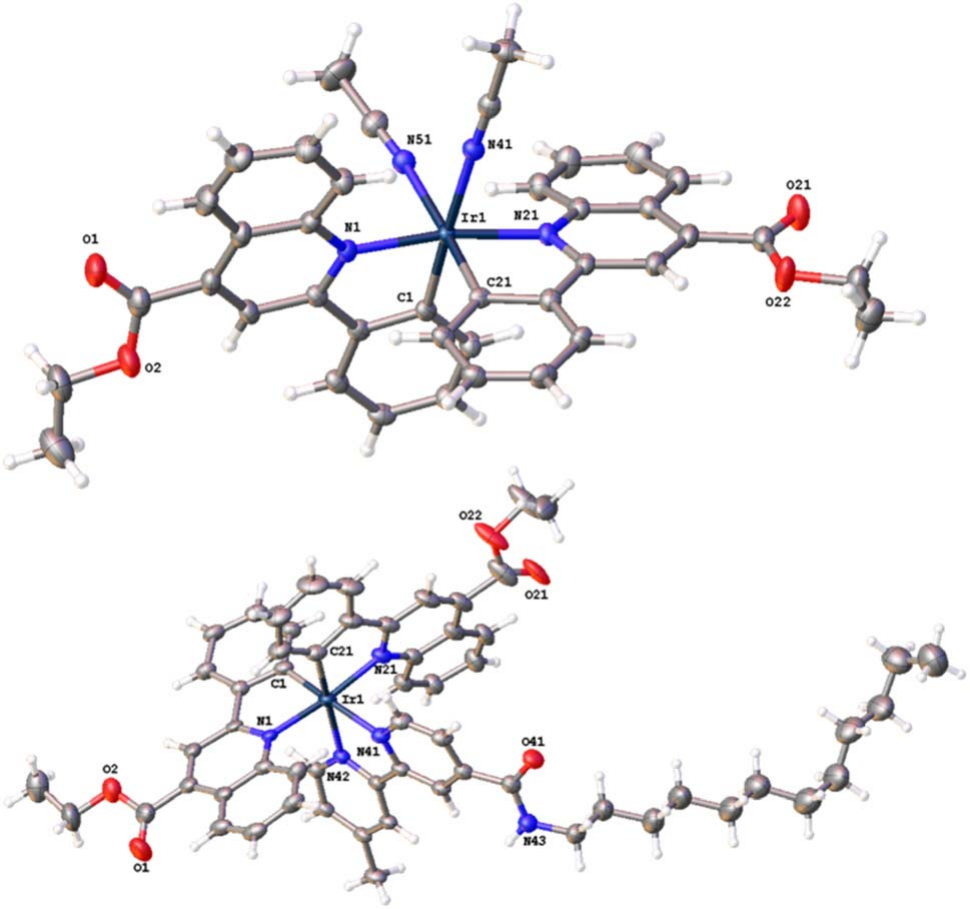
Top: The crystal structure of *cis*-[Ir(epqc)_2_(MeCN)_2_]BF_4_ (disordered counter ion and ether solvent omitted for clarity). Bottom: The crystal structure of [Ir(epqc)_2_(L^3^)]BF_4_ (counter ion and disorder (ligand and solvent ether) omitted for clarity).

Bright red-coloured crystals of *cis*-[Ir(epqc)_2_(MeCN)_2_]BF_4_ and [Ir(epqc)_2_(L^3^)]BF_4_ were obtained for X-ray diffraction studies *via* recrystallisation from a concentrated dichloromethane solution of the complex followed by vapour diffusion of diethyl ether. Data collection parameters are shown in the ESI (Tables S1 and S2†), together with bond length and bond angle data (Table 1). The resultant structures are shown in Fig. 2. The X-ray structures show that both complexes adopt a distorted octahedral geometry. In both cases, the cyclometalating ligands retain the *cis*-C, *trans*-N coordination of the chloro-bridged dimer precursor. For *cis*-[Ir(epqc)_2_(MeCN)_2_]BF_4_ the coordinated MeCN ligands are mutually *cis*. For [Ir(epqc)_2_(L^3^)]BF_4_ the bipyridine ligand is coordinated *trans* to the cyclometalated phenyl rings with Ir–N bond lengths (2.16 Å) slightly longer than those of the epqc ligands (2.09 Å). These parameters are in good agreement with those of related Ir(iii) complexes with a similar coordination sphere and ligand environment.^24^ It is also noteworthy that in the structure of [Ir(epqc)_2_(L^3^)]BF_4_ there was an absence of disorder within the extended *n*-dodecyl chain. Within the crystal packing data, much of the dodecyl chain lies within the groove created between the two epqc ligands of a neighbouring complex, with the end lying along the opposite side of another epqc ligand of a third complex. The disordered solvent ether slots between the neighbouring complexes leaving little space for interaction between neighbouring dodecyl chains.

**Table 1:** Selected bond lengths (Å) obtained from the X-ray structures

*cis*-[Ir(epqc)_2_(MeCN)_2_]BF_4_		[Ir(epqc)_2_(L^3^)]BF_4_	
Ir1–C1	1.991(3)	Ir1–C1	1.942(11)
Ir1–C21	1.997(3)	Ir1–C21	2.005(3)
Ir1–N21	2.089(2)	Ir1–C1B	2.11(3)
Ir1–N1	2.092(2)	Ir1–N1	2.082(8)
Ir1–N41	2.140(2)	Ir1–N1B	2.10(2)
Ir1–N51	2.150(2)	Ir1–N21	2.097(3)
		Ir1–N42	2.163(2)
		Ir1–N41	2.166(3)

### Redox and electronic properties

The electrochemical characteristics of the ester functionalised complexes [Ir(emptz)_2_(L)]BF_4_ and [Ir(epqc)_2_(L)]BF_4_ were investigated in deoxygenated dichloromethane. The cyclic voltammograms, measured at a platinum disc electrode (scan rate 200 mV s^−1^, 1 × 10^−3^ M solutions, 0.1 M [NBu_4_][PF_6_] as a supporting electrolyte) generally showed one non-fully reversible oxidation around +1.57 V (where C^N = epqc) and +1.44 V (where C^N = emptz) which were assigned to the Ir^3+/4+^ couple. The data for the current series of complexes is in very good agreement with previously reported systems, [Ir(emptz)_2_(bipy)]PF_6_ and [Ir(epqc)_2_(bipy)]PF_6_ which incorporate an unsubstituted bipyridine ligand.^24^ The Ir^3+/4+^ oxidation potentials are slightly higher for the emptz variants *versus* epqc species, suggesting that the Ir^3+^ state is relatively more stabilised in the former. As expected, within each C^N grouping of L^1–3^ complexes there is little variation in oxidation potential as a function of alkyl chain length. The complexes also showed a fully or partially reversible reduction wave around −1.22 V which are assigned to ligand-centred processes involving the bipyridine ligands, but with the potential for some contribution from the cyclometalating ligands.

UV-Vis absorption spectra and corresponding data were obtained using aerated MeCN solutions (10^−5^ M). The free bipyridine ligands (L^1–3^) possess *λ*_max_ ∼ 280 nm (π–π*) and do not absorb beyond 320 nm. As expected, the length of the alkyl chain had negligible effect on the absorption properties of L^1–3^. Free epqcH and emptzH also show strong absorption bands in the UV region assigned to a combination of π–π* transitions within the different aromatic moieties (Fig. S31 and S32†). The corresponding Ir(iii) complexes show a combination of the intense, spin-allowed, ligand-centred transitions (bathochromically-shifted upon metal coordination) and weaker bands at 470 nm (*ε* ∼ 2700 M^−1^ cm^−1^) and 435 nm (*ε* ∼ 8000 M^−1^ cm^−1^) for [Ir(epqc)_2_(L^3^)]BF_4_ and [Ir(emptz)_2_(L^3^)]BF_4_, respectively. These latter bands were assigned to spin-allowed metal-to-ligand charge transfer (^1^MLCT) transitions and correlate well with those reported for related complexes with comparable ligand structures.^24^ The MLCT bands tail well into the visible region and prior studies, supported by computational work, have shown the possibility of spin forbidden ^3^MLCT transitions contributing at these longer wavelengths (Fig. S31 and S32†).^25^

Room temperature luminescence measurements (Table 2, Fig. S33 and S34†) were carried out using MeCN solutions (10^−5^ M) of the complexes. The emission spectra of [Ir(epqc)_2_(L^1–3^)]BF_4_ typically revealed a broad emission peak around 630 nm. The corresponding lifetimes were obtained by fitting the decay kinetics (*λ*_ex_ = 295 nm) to a monoexponential function. The different lifetime values (176–189 ns) are typically within error (±10%) and consistent with a ^3^MLCT emitting state, as noted previously.^24^ Further low temperature measurements (measured at 77 K on a 1 : 1 EtOH/MeOH glass) revealed a hypsochromically shifted peak (typical of a matrix induced rigidochromism) at *ca.* 600 nm with a moderate vibronic structure. These emission characteristics were retained across the three [Ir(epqc)_2_(L^1–3^)]BF_4_ complexes; the variation in alkyl chain length had a negligible effect on the photophysical properties.

**Table 2:** Luminescence properties of the iridium complexes^a^

Complex	*λ*_em_ b/nm	*τ* c/ns	*Φ* d (%)
[Ir(epqc)_2_(L^1^)]BF_4_	631 (559)	186	3
[Ir(epqc)_2_(L^2^)]BF_4_	630 (560)	176	2
[Ir(epqc)_2_(L^3^)]BF_4_	630 (560)	189	1
[Ir(emptz)_2_(L^1^)]BF_4_	550 (539)	238	2
[Ir(emptz)_2_(L^2^)]BF_4_	550 (540)	263	2
[Ir(emptz)_2_(L^3^)]BF_4_	554 (534)	199	2

a Measurements obtained on aerated 10^−5^ M MeCN solutions unless otherwise stated.

b *λ*_ex_ = 450 nm; values in parenthesis are emission maxima from EtOH–MeOH (1 : 1) glass at 77 K.

c *λ*_ex_ = 295 or 459 nm.

d [Ru(bpy)_3_](PF_6_)_2_ as reference of 1.8% in aerated MeCN.

**Fig. 3:**
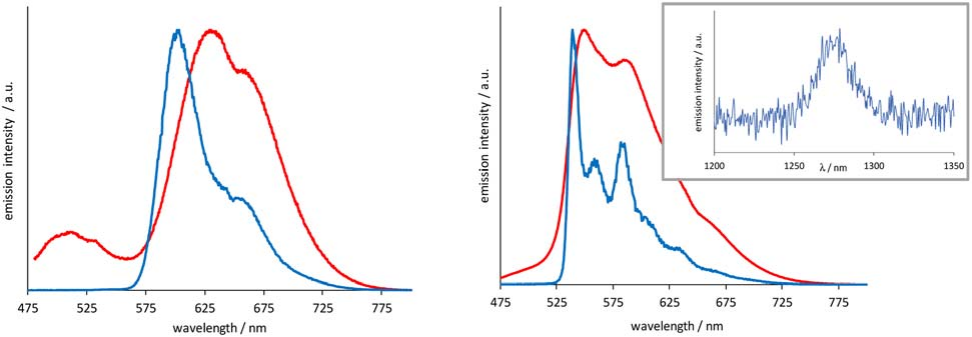
Normalized emission spectra (*λ*_ex_ = 450 nm) recorded at 293 K (red) and 77 K (blue) for [Ir(epqc)_2_(L^3^)]BF_4_ (left) and [Ir(emptz)_2_(L^3^)]BF_4_ (right). Near-IR emission spectrum of [Ir(emptz)_2_(L^3^)]BF_4_ in aerated MeCN showing ^1^Δ_g_ → ^3^Σ_g^−^_ transition.

The [Ir(emptz)_2_(L^1–3^)]BF_4_ complexes revealed a dominant emission peak *ca.* 560 nm with much more pronounced vibronic features (Fig. 3) compared to [Ir(epqc)_2_(L^1–3^)]BF_4_. The corresponding lifetimes were in the range of 199–263 ns and again consistent with an emitting state of triplet character. The low temperature spectra (77 K, MeOH–EtOH (1 : 1) glass) showed a highly structured emission profile perhaps indicative of a stronger ligand-centred contribution (^3^LC) to the character of the emitting state or strong coupling of ligand vibrational states with the ^3^MLCT state.^24^ The triplet excited state character of these complexes was further probed using steady state near-IR emission spectroscopy. Following irradiation of the spin allowed ^1^MLCT bands using 400–450 nm in aerated MeCN, selected complexes showed a weak emission peak at ∼1270 nm, which was assigned to the characteristic emission (^1^Δ_g_ → ^3^Σ_g^−^_) from photogenerated ^1^O_2_ (see inset Fig. 3).

### Deprotection of the complexes and microemulsion studies

**Scheme 3:**
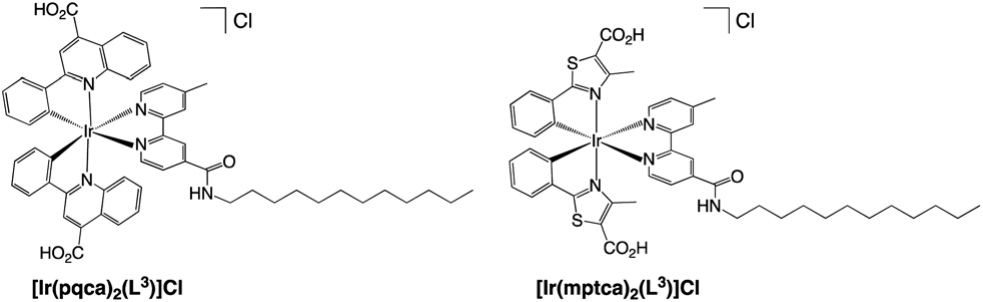
Structures of the deprotected amphiphilic iridium(iii) complexes synthesised in this study.

The dodecyl alkyl chain functionalised Ir(iii) complexes were selected for further studies and optimised compatibility with the chosen microemulsion material based on a dodecyl chain functionalised, *N*-methyl imidazolium salt. Firstly, the complexes were treated to facilitate deprotection of the ester groups which was achieved using a mixture of acetone and aqueous KOH (1 M) affording the carboxylic acid functionalised complexes as their chloride salts, [Ir(pqca)_2_(L^3^)]Cl and [Ir(mptca)_2_(L^3^)]Cl (where pqca = 2-phenyl-quinoline-4-carboxylic acid, and mptca = 4-methyl-2-phenylthiazole-5-carboxylic acid) (Scheme 3). The deprotection was confirmed through the absence of the ethyl protons in the ^1^H NMR spectra of [Ir(pqca)_2_(L^3^)]Cl and [Ir(mptca)_2_(L^3^)]Cl. All of the complexes were also characterised *via* high resolution mass spectrometry, which exhibited the parent cation peak cluster for with the signature iridium isotope pattern (Fig. S26–S30†).

From a photophysical perspective, the deprotected complexes [Ir(mptca)_2_(L^3^)]Cl and [Ir(pqca)_2_(L^3^)]Cl demonstrated sufficient solubility to allow investigation in both MeCN and aqueous solvent. The CO_2_H group on the backbone of the cyclometalating ligand is likely to be deprotonated around neutral pH (for quinoline-4-carboxylic acid the p*K*_a_ ∼ 4.5)^26^ and therefore a zwitterion or negative overall charge is possible for these complexes in water. The emission maximum of [Ir(pqca)_2_(L^3^)]Cl in MeCN was 593 nm (showing the influence of changing CO_2_Et to CO_2_H) which was bathochromically shifted in water to 623 nm (*τ* = 332 ns). For [Ir(mptca)_2_(L^3^)]Cl the emission appeared as a broadened peak with a maximum in MeCN of 580 nm which was bathochromically shifted in water to 586 nm (*τ* = 173 ns). These data (Table 3) show that the phosphorescent character of the complexes is retained after deprotection.

**Table 3:** Luminescence properties of the deprotected iridium complexes^a^

Complex	*λ*_em_ b/nm	*τ* c/ns	*Φ* d (%)
**[Ir(pqca)**_**2**_ **(L** ^**3**^ **)]Cl**			
In MeCN	593	230	3
In water	623	332	—

**[Ir(mptca)**_**2**_ **(L** ^**3**^ **)]Cl**			
In MeCN	580	270	2
In water	586	173	—

a Measurements obtained on aerated 10^−5^ M solutions.

b *λ*_ex_ = 450 nm.

c *λ*_ex_ = 295 or 459 nm.

d Using [Ru(bipy)_3_](PF_6_)_2_ as reference (1.8% in aerated MeCN).

**Scheme 4:**
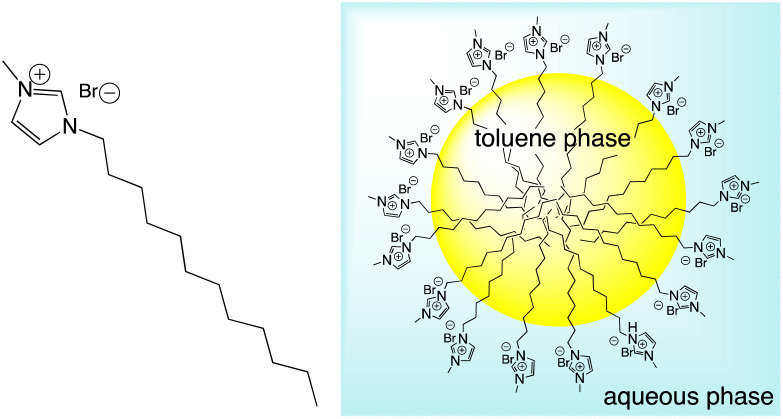
Structure (left) of the *N*-methyl C12 substituted imidazolium salt, [MeImC_12_]Br, used in the formulation of oil-in-water microemulsions (shown right).

To investigate the behaviour of luminescent Ir(iii) complexes in a microemulsion, the parent microemulsion system was firstly demonstrated to be an aggregation colloid. The microemulsion was formulated (see Experimental section) from a dodecyl chain functionalised, *N*-methyl imidazolium salt, [MeImC_12_]Br, *n*-butanol and water, and was found to be effective at solubilising small to moderate volumes of toluene as an oil-in-water microemulsion (Scheme 4).

**Fig. 4:**
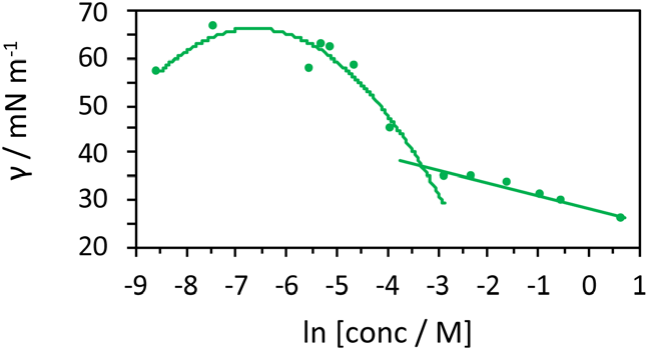
Tensiometric data for [MeImC_12_]Br/^*n*^BuOH/H_2_O (microemulsion) measured in ultra-pure water at room temperature. CMC (±0.1 mM) was obtained as 36.5 mM.

As the components are in an aqueous environment it is assumed that they orientate with the lipophilic dodecyl chains towards the interior of the micelle and thus positioned away from bulk water (shown schematically in Scheme 4). Tensiometry studies provided the critical micelle concentration for the carrier [MeImC_12_]Br system of 36.5 mM (Fig. 4).

**Fig. 5:**
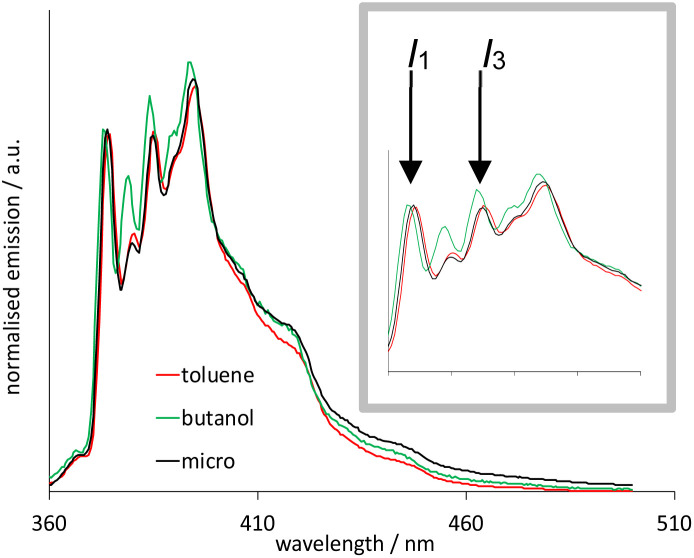
Emission spectra (*λ*_ex_ = 340 nm) of 1 μM pyrene in toluene (red), *n*-butanol (green) and microemulsion (black; [MeImC_12_]Br/^*n*^BuOH/toluene/H_2_O) at room temperature. Expansion of the ratiometric *I*_1_/*I*_3_ vibronic features shown inset.

In this study, 10 wt% toluene was found to be freely soluble in this system, and it is noteworthy that this microemulsion is particularly effective at solubilising low polarity organic materials. To verify that this system was a microemulsion, we exploited the polarity dependent vibronic features of pyrene fluorescence.^27^Fig. 5 illustrates the fluorescence emission spectra of pyrene (*λ*_ex_ = 340 nm) obtained in aerated toluene, *n*-butanol and microemulsion micromolar solutions. Firstly, the spectra show pyrene monomer emission in each case, with no evidence of a bathochromically shifted excimer band, as expected for this concentration regime. Whilst the spectra are comparable, closer inspection of the first and third vibronic bands (*I*_1_ and *I*_3_, respectively) reveal a subtly differing ratio of intensities. These vibronic features are well known to be sensitive to the local polarity of the medium.^28^ While pyrene has very poor solubility in water, the reported *I*_3_ : *I*_1_ value is 0.64 which is much lower than for hydrocarbon or aromatic solvents, such as *n*-butanol and toluene.^28^

It is obvious that the spectral profiles for the toluene and microemulsion samples are essentially identical, whilst that of the *n*-butanol solution is different. Since the organic loading of this system significantly exceeds the solubility of toluene in water, this is strongly indicative of a microemulsion: the pyrene is solubilised within the toluene-rich core of the microemulsion droplets which are, in turn, dispersed in an aqueous medium (Scheme 4).

**Fig. 6:**
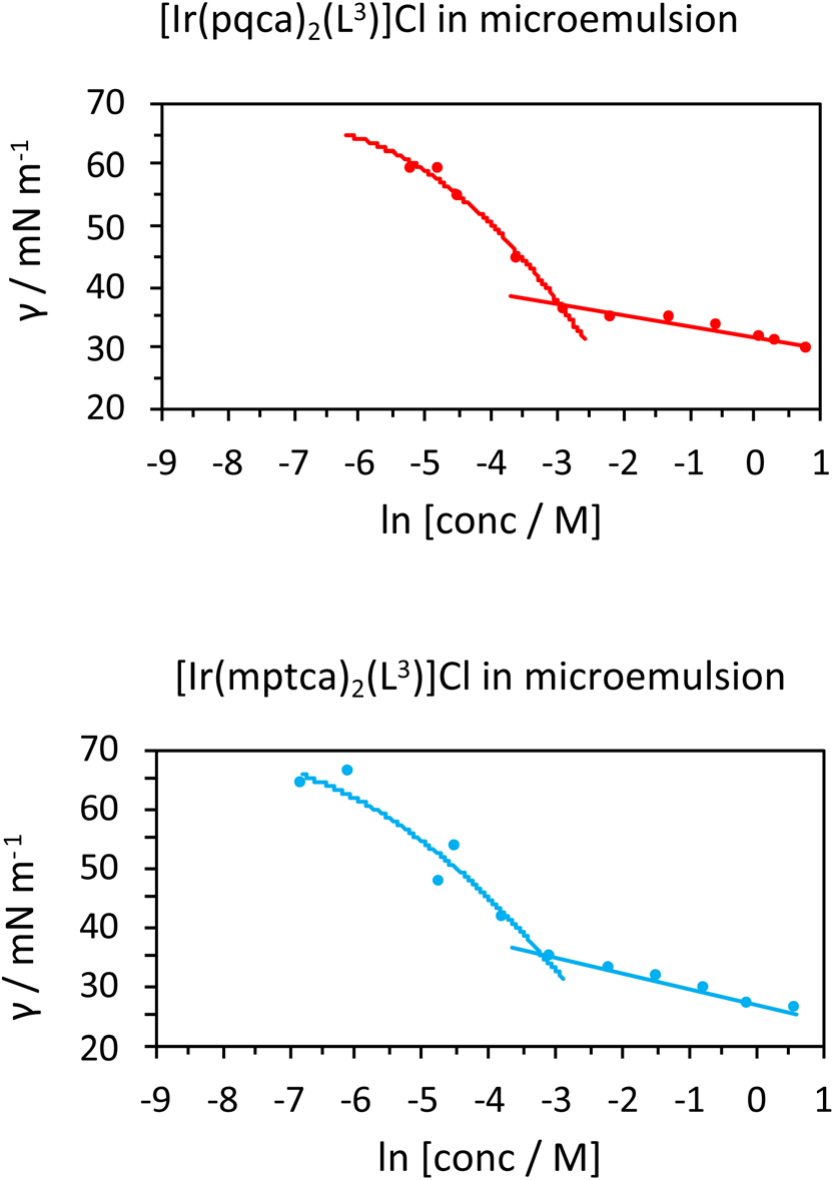
Tensiometric data measured in ultra-pure water at room temperature. CMCs (±0.1 mM) obtained as 51.3 mM for [Ir(pqca)_2_(L^3^)]Cl in microemulsion and 40.4 mM for [Ir(mptca)_2_(L^3^)]Cl in microemulsion.

Initial studies on [Ir(pqca)_2_(L^3^)]Cl and [Ir(mptca)_2_(L^3^)]Cl showed that while these complexes were soluble in water, it was insufficient to form micelles in their own right, *i.e.* the CMC lies at a point where the complex is no longer soluble in water. This contrasts with previous work by Bowers *et al.* on alkyl chain functionalised [Ru(bipy)_2_(*p*,*p*′-dialkyl-2,2′-bipy)]Cl_2_ complexes which show aggregation and CMC behaviour as metallosurfactants and presumably benefit from their dicationic nature.^29^ Therefore, having established the micellar properties of the microemulsion, [Ir(pqca)_2_(L^3^)]Cl and [Ir(mptca)_2_(L^3^)]Cl were investigated as dopants into the carrier micellar system [MeImC_12_]Br/^*n*^BuOH/H_2_O using 2 wt% of each Ir(iii) complex. The resultant coloured solutions were capable of solubilising at least 10 wt% toluene to give stable microemulsions at room temperature. The resultant tensiometry plots (Fig. 6) confirmed the incorporation of the Ir(iii) amphiphiles into the micellar system. Clear CMC points were obtained for each system with pleasing linearity in the post-CMC region of the plot.

From the surface tension plots, it was clear that the presence of the Ir(iii) complexes raises the CMC of the micellar system, suggesting slightly less favourable conditions for formation of micelles when the [MeImC_12_]Br/^*n*^BuOH/H_2_O system incorporates an Ir(iii) complex. This may be due to a number of factors such as the larger steric hindrance of the complex head group or the disruption of the cationic headgroups of the imidazolium units as previously noted in metallosurfactants.^30^ Interestingly, [Ir(pqca)_2_(L^3^)]Cl has the higher CMC value (51.3 mM *vs.* 40.4 mM) of the two complexes investigated.

**Fig. 7:**
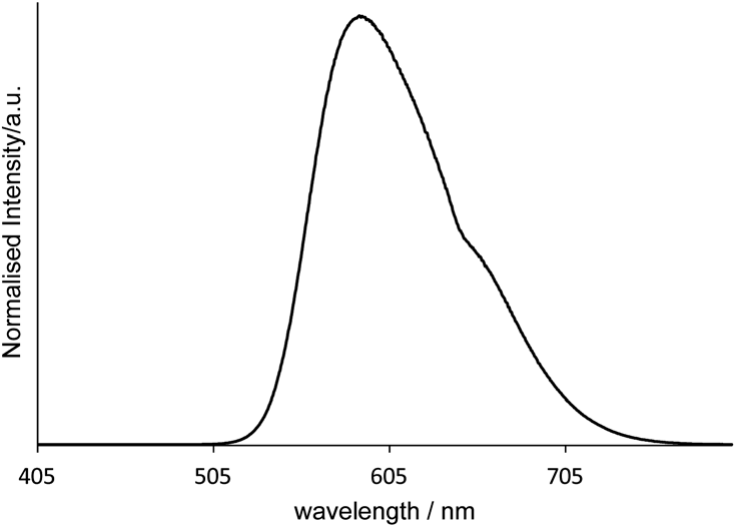
Emission spectrum (*λ*_ex_ = 350 nm) of a [MeImC_12_]Br/^*n*^BuOH/H_2_O microemulsion assembly doped with [Ir(pqca)_2_(L^3^)]Cl measured in the post-CMC region in ultra-pure water at room temperature.

Finally, Fig. 7 shows the emission spectrum for [Ir(pqca)_2_(L^3^)]Cl when doped into the microemulsion at 2 wt% (*i.e.* at a concentration above the CMC). The photophysical properties (*λ*_em_ = 593 nm; observed lifetime = 210 ns) are clearly consistent with the retention of the luminescent character of the dopant. While further studies are required to fully elucidate the doped micelle structures, it seems likely that in water the Ir(iii) complexes arrange in a way that places the lipophilic dodecyl chains orienting towards the hydrophobic interior of the micelle.

## Conclusions

Alkyl chain functionalised luminescent Ir(iii) complexes can be synthesised to incorporate both hydrophilic and hydrophobic moieties. In this study we have shown that the long-lived, visible luminescence of dodecyl-conjugated Ir(iii) organometallics can be combined with a dodecyl imidazolium salt carrier system to produce luminescent, oil-in-water microemulsion assemblies. The micellar structure of the carrier system was probed using the sensitive fluorescence properties of pyrene, but further studies are required to determine the size and shape of the doped microemulsion assemblies. Future work should consider the design of the luminescent complexes which could be altered to increase the cationic charge and thus hydrophilicity of the ‘head group’. This could feasibly be achieved by considering the use of alkyl ammonium substituents that can be appended to the (cyclometalating) ligands.

## Experimental

All reagents and solvents were commercially available and were used without further purification if not stated otherwise. 4-Methyl-2,2′-bipyridine-4′-carboxylic acid, 4-methyl-2,2′-bipyridine-4′-carbonyl chloride, ethyl-2-phenylquinoline-4-carboxylate (epqcH), ethyl-methyl-2-phenylthiazole-5-carboxylate (emptzH), [Ir(epqc)_2_(μ-Cl)Ir(epqc)_2_] and [Ir(emptz)_2_(μ-Cl)Ir(emptz)_2_] were all synthesised according to previous methods.^24^ For the measurement of ^1^H and ^13^C NMR spectra a Bruker Fourier (250, 300 MHz), 400 UltraShield™ (400 MHz) or Ascend™500 (500 MHz) was used. The obtained chemical shifts *δ* are reported in ppm and are referenced to the residual solvent signal. Spin–spin coupling constants *J* are given in Hz. Low-resolution mass spectra were obtained by the staff at Cardiff University. High-resolution mass spectra were carried out at the EPSRC National Mass Spectrometry Facility at Swansea University. High resolution mass spectral (HRMS) data were obtained on a Waters MALDI-TOF mx at Cardiff University or on a Thermo Scientific LTQ Orbitrap XL by the EPSRC UK National Mass Spectrometry Facility at Swansea University. IR spectra were obtained from a Shimadzu IR-Affinity-1S FTIR. UV-Vis studies were performed on a Jasco V-570 spectrophotometer as MeCN solutions (2.5 or 5 × 10^−5^ M). Photophysical data were obtained on a JobinYvon-Horiba Fluorolog spectrometer fitted with a JY TBX picosecond photodetection module as MeCN solutions. The pulsed source was a Nano-LED configured for 459 nm output operating at 1 MHz. Luminescence lifetime profiles were obtained using the JobinYvon-Horiba FluoroHub single photon counting module and the data fits yielded the lifetime values using the provided DAS6 deconvolution software. Quantum yield measurements were obtained on aerated MeCN solutions of the complexes using [Ru(bpy)_3_](PF_6_)_2_ in aerated MeCN as a standard (*Φ* = 0.018).^31^

Electrochemical studies were carried out using a Parstat 2273 potentiostat in conjunction with a three-electrode cell. The auxiliary electrode was a platinum wire and the working electrode a platinum (1.0 mm diameter) disc. The reference was a silver wire separated from the test solution by a fine porosity frit and an agar bridge saturated with KCl. Solutions (10 mL CH_2_Cl_2_) were 1.0 × 10^−3^ mol dm^−3^ in the test compound and 0.1 mol dm^−3^ in [NBu_4_][PF_6_] as the supporting electrolyte. Under these conditions, *E*_0_′ for the one-electron oxidation of [Fe(η-C_5_H_5_)_2_], added to the test solutions as an internal calibrant, is +0.46 V. Unless specified, all electrochemical values are at *ν* = 200 mV s^−1^.

### X-ray crystallography

#### Data collection and processing

For both samples, a suitable crystal^32^ was selected and mounted on a MITIGEN holder in oil on a Rigaku FRE+ (45.0 kV, 55.0 mA) equipped with VHF Varimax confocal mirrors (70 μm focus) and an AFC12 goniometer and HG Saturn 724+ detector diffractometer. The crystals were kept at *T* = 100(2) K during data collection. Data were measured using profile data from ω-scans using MoK_α_ radiation. Cell determination and data collection were carried out using CrystalClear.^33^ With the data reduction, cell refinement and absorption correction using CrystalisPro.^34^ Using Olex2,^35^ the structures were solved with the ShelXT^36^ structure solution program and the models were refined with version 2018/3 of ShelXL^37^ using Least Squares minimisation. All non-hydrogen atoms were refined anisotropically. Hydrogen atom positions were calculated geometrically and refined using the riding model.

For sample *cis*-[Ir(epqc)_2_(MeCN)_2_]BF_4_, both the BF_4_ anion and ether molecule are disordered over two positions. As such various geometrical (SAME, SADI) and displacement (SIMU, RIGU) restraints were applied.

For sample [Ir(epqc)_2_(L^3^)]BF_4_, there is disorder of the ethyl ester groups. This leads to whole molecule disorder for one of the ligands. Due to this various geometrical (SAME, SADI) and displacement (RIGU) restraints were employed. Also, there is a molecule of disordered ether lying over an inversion centre. To model this, geometric (AFIX) constraints and displacement restraints (SIMU, RIGU) were applied to its atoms.

Crystal data for *cis*-[Ir(epqc)_2_(MeCN)_2_]BF_4_. C_44_H_44_BF_4_IrN_4_O_5_, *M*_r_ = 987.84, triclinic, *P*1̄ (No. 2), *a* = 8.7992(2) Å, *b* = 16.6146(4) Å, *c* = 16.7086(4) Å, *α* = 119.195(2)°, *β* = 91.698(2)°, *γ* = 102.782(2)°, *V* = 2052.24(9) Å^3^, *T* = 100(2) K, *Z* = 2, *Z*′ = 1, *μ*(Mo K_α_) = 3.322 mm^−1^, 40 539 reflections measured, 9389 unique (*R*_int_ = 0.0351) which were used in all calculations. The final w*R*_2_ was 0.0674 (all data) and *R*_1_ was 0.0264 (*I* ≥ 2*σ*(*I*)).

Crystal data for [Ir(epqc)_2_(L^3^)]BF_4_. C_62_H_68_BF_4_IrN_5_O_5.5_, *M*_r_ = 1250.22, triclinic, *P*1̄ (No. 2), *a* = 9.7337(2) Å, *b* = 16.3240(3) Å, *c* = 18.6845(4) Å, *α* = 100.368(2)°, *β* = 91.400(2)°, *γ* = 104.629(2)°, *V* = 2818.06(10) Å^3^, *T* = 100(2) K, *Z* = 2, *Z*′ = 1, *μ*(Mo K_α_) = 2.437 mm^−1^, 48 362 reflections measured, 12 877 unique (*R*_int_ = 0.0285) which were used in all calculations. The final w*R*_2_ was 0.0781 (all data) and *R*_1_ was 0.0310 (*I* ≥ 2*σ*(*I*)).

### Synthesis

#### *N*-Octyl-4′-methyl-2,2′-bipyridine-4-carboxamide (L^1^)

4′-Methyl-2,2′-bipyridine-4-carbonyl chloride (0.47 g, 2.02 mmol) and 1-octylamine (0.37 g, 2.86 mmol) were dissolved in MeCN (5 mL) with *N*,*N*-diisopropylamine (1.20 mL, 6.89 mmol) in MeCN (5 mL) and stirred at 60 °C for 24 hours under a N_2_ atmosphere. The solvent was removed *in vacuo* and the crude product dissolved in DCM, washed with water and dried over MgSO_4_. Solvent was removed *in vacuo* to give the title compound as a light brown solid. Yield: 0.2247 g, 0.69 mmol, 34%. ^1^H NMR (400 MHz, CDCl_3_): *δ*_H_ = 8.76 (1H, d, ^3^*J*_HH_ = 5.0 Hz), 8.57 (1H, s), 8.50 (1H, d, ^3^*J*_HH_ = 5.0 Hz), 8.24 (1H, s), 7.75 (1H, app. dd, *J*_HH_ = 1.7, 5.0 Hz), 7.16–7.14 (1H, app. m), 6.67–6.59 (1H, br. s, N*H*), 3.44 (2H, q, ^3^*J*_HH_ = 6.8 Hz, C*H*_2_), 2.43 (3H, s, C*H*_3_), 1.62–1.58 (2H, m, C*H*_2_), 1.35–1.25 (10H, br. s, C*H*_2_), 0.85 (3H, t, ^3^*J*_HH_ = 6.8 Hz, C*H*_3_) ppm. ^13^C{^1^H} NMR (100 MHz, CDCl_3_): *δ*_C_ = 165.7 (*C*O), 156.9, 155.1, 150.0, 148.9, 148.5, 143.0, 125.2, 122.2, 121.8, 117.5, 40.4, 31.8, 29.5, 29.2, 27.0, 22.7, 21.2, 14.1 ppm. LRMS (ES^+^) found *m*/*z* 326.22, calcd 326.22 for [M + H]^+^; HRMS (ES^+^) found *m*/*z* 326.2227, calcd 326.2226 for [C_20_H_28_N_3_O]^+^. UV/Vis (CH_3_CN): *λ*_max_/nm (*ε*/M^−1^ cm^−1^) = 284 (9500), 250 sh. (8900), 243 (9200), 208 (13 600). IR (solid): *ν* 3302 (N–H), 2922, 2939, 2846, 1629 (C=O), 1525, 1257 cm^−1^.

#### *N*-Decyl-4′-methyl-2,2′-bipyridine-4-carboxamide (L^2^)

As for L^1^ but using 4′-methyl-2,2′-bipyridine-4-carbonyl chloride (0.44 g, 1.89 mmol), 1-decylamine (0.42 mL, 2.10 mmol) and *N*,*N*-diisopropylethylamine (1.12 mL, 6.43 mmol) in MeCN (5 mL) to give the title compound as a light brown solid. Yield: 0.2040 g, 0.58 mmol, 31%. ^1^H NMR (400 MHz, CDCl_3_): *δ*_H_ = 8.74 (1H, d, ^3^*J*_HH_ = 5.0 Hz), 8.57 (1H, s), 8.49 (1H, d, ^3^*J*_HH_ = 5.0 Hz), 8.22 (1H, s), 7.74 (1H, app. dd, *J*_HH_ = 1.7, 5.0 Hz), 7.13 (1H, d, ^3^*J*_HH_ = 4.2 Hz), 6.76–6.73 (1H, br. s, N*H*), 3.43 (2H, q, ^3^*J*_HH_ = 6.8 Hz, C*H*_2_), 2.43 (3H, s, C*H*_3_), 1.59 (2H, app. t, ^3^*J*_HH_ = 7.1 Hz, C*H*_2_), 1.33–1.23 (14H, br. m, C*H*_2_), 0.85 (3H, t, ^3^*J*_HH_ = 6.8 Hz, C*H*_3_) ppm. ^13^C{^1^H} NMR (100 MHz, CDCl_3_): *δ*_C_ = 165.8 (*C*O), 156.9, 155.2, 150.1, 149.0, 148.6, 143.0, 125.3, 122.3, 122.0, 117.4, 41.1, 40.4, 36.2, 32.0, 30.5, 29.6, 29.4, 27.1, 22.8, 21.3, 14.2 ppm. LRMS (ES^+^) found *m*/*z* 354.26, calcd 354.25 for [M + H]^+^; HRMS (ES^+^) found *m*/*z* 354.2538, calcd 354.2538 for [C_22_H_32_N_3_O]^+^. UV/Vis (CH_3_CN): *λ*_max_/nm (*ε*/M^−1^ cm^−1^) = 277 (23 000), 250 (16 000), 245 sh. (14 900), 206 (16 900). IR (solid): *ν* 3304 (N–H), 2954, 2920, 2848, 1629 (C=O), 1525, 1267 cm^−1^.

#### *N*-Dodecyl-4′-methyl-2,2′-bipyridine-4-carboxamide (L^3^)

As for L^1^ but using 4′-methyl-2,2′-bipyridine-4-carbonyl chloride (0.19 g, 0.82 mmol), 1-dodecylamine (0.15 g, 0.81 mmol) and *N*,*N*-diisopropylethylamine (0.48 mL, 2.76 mmol) to give the title compound as a light brown solid. Yield: 0.1018 g, 0.27 mmol, 33%. ^1^H NMR (400 MHz, CDCl_3_): *δ*_H_ = 8.75 (1H, d, ^3^*J*_HH_ = 5.0 Hz), 8.57 (1H, s), 8.49 (1H, d, ^3^*J*_HH_ = 5.0 Hz), 8.23 (1H, s), 7.75 (1H, dd, ^3^*J*_HH_ = 1.7, 5.0 Hz), 7.15 (1H, dd, ^3^*J*_HH_ = 0.6, 4.9 Hz), 6.76–6.72 (1H, m, N*H*), 3.48 (2H, q, ^3^*J*_HH_ = 6.2 Hz, C*H*_2_), 2.85 (3H, s, C*H*_3_), 1.69–1.60 (2H, m, C*H*_2_), 1.39–1.15 (18H, br. m, C*H*_2_), 0.85 (3H, t, ^3^*J*_HH_ = 6.8 Hz, C*H*_3_) ppm. ^13^C{^1^H} NMR (100 MHz, CDCl_3_): *δ*_C_ = 165.6 (*C*O), 156.7, 155.1, 150.3, 149.0, 148.9, 143.2, 125.4, 122.6, 122.2, 117.6, 40.4, 32.0, 29.8, 29.7, 29.6, 29.5, 29.4, 27.1, 22.8, 21.4, 14.2 ppm. LRMS (ES^+^) found *m*/*z* 382.29, calcd 382.29 for [M + H]^+^; HRMS (ES^+^) found *m*/*z* 382.2851, calcd 382.2850 for [C_24_H_36_N_3_O]^+^. UV/Vis (CH_3_CN): *λ*_max_/nm (*ε*/M^−1^ cm^−1^) = 278 (19 400), 250 (14 200), 245 sh. (13 300), 206 (15 800). IR (solid): *ν* 3305 (N–H), 2939, 2918, 2848, 1631 (C=O), 1525, 1257 cm^−1^.

#### [Ir(epqc)_2_(MeCN)_2_](BF_4_)

AgBF_4_ (0.02 g, 0.10 mmol) in MeCN (10 mL) was added to [Ir(epqc)_2_(μ-Cl)_2_Ir(epqc)_2_] (0.08 g, 0.05 mmol) in MeCN (25 mL) and the solution heated to reflux for 2 hours under a N_2_ atmosphere. The solvent was removed *in vacuo* and the product precipitated with DCM/Et_2_O to give the title compound as a red crystalline solid. Yield: 0.0600 g, 0.06 mmol, 64%. ^1^H NMR (400 MHz, CDCl_3_): *δ*_H_ = 9.12 (2H, d, ^3^*J*_HH_ = 8.9 Hz), 8.72 (2H, d, ^3^*J*_HH_ = 8.4 Hz), 8.35 (2H, s), 8.05–7.95 (2H, m), 7.72 (2H, app. t, ^3^*J*_HH_ = 7.7 Hz), 7.60 (2H, d, ^3^*J*_HH_ = 7.7 Hz), 6.87 (2H, app. t, ^3^*J*_HH_ = 7.4 Hz), 6.64 (2H, app. t, ^3^*J*_HH_ = 7.5 Hz), 5.70 (2H, d, ^3^*J*_HH_ = 7.2 Hz), 4.58 (4H, q, ^3^*J*_HH_ = 7.2 Hz, C*H*_2_), 2.24 (6H, s, C*H*_3_CN), 1.51 (6H, t, ^3^*J*_HH_ = 7.2 Hz, C*H*_3_) ppm. ^13^C{^1^H} NMR (125 MHz, CDCl_3_): *δ*_C_ = 165.5 (*C*O), 149.1, 145.9, 132.7, 131.4, 130.4, 128.6, 128.4, 127.0, 126.5, 126.3, 124.8, 122.3, 118.1, 70.1, 65.9, 63.4, 62.8, 59.2, 14.7, 3.5 ppm. HRMS (ES^+^) found *m*/*z* 745.1664, calcd 745.1671 for [IrC_36_H_28_N_2_O_4_]^+^. UV/Vis (CH_3_CN): *λ*_max_/nm (*ε*/M^−1^ cm^−1^) = 313 (29 300), 273 (59 000), 234 (67 400). IR (solid): *ν* 2980, 2962, 2372, 2311 (C≡N), 1716 (C=O), 1541, 1375, 1261, 1242, 1056, 1016 br (B–F), 792, 761 cm^−1^.

#### [Ir(emptz)_2_(MeCN)_2_](BF_4_)

Made similarly to [Ir(epqc)_2_(MeCN)_2_](BF_4_) but using [Ir(emptz)_2_(μ-Cl)_2_Ir(emptz)_2_] (0.10 g, 0.07 mmol) in MeCN (25 mL) with AgBF_4_ (0.03 g, 0.15 mmol) in MeCN (10 mL) to give the title compound as an orange crystalline solid. Yield: 0.0894 g, 0.10 mmol, 75%. ^1^H NMR (400 MHz, CDCl_3_): *δ*_H_ = 7.53 (2H, d, ^3^*J*_HH_ = 5.6 Hz), 6.94 (2H, app. t, ^3^*J*_HH_ = 6.0 Hz), 6.84 (2H, app. t, ^3^*J*_HH_ = 8.0 Hz), 6.22 (2H, d, ^3^*J*_HH_ = 7.4 Hz), 4.47 (4H, q, ^3^*J*_HH_ = 5.8 Hz, C*H*_2_), 3.08 (6H, s, C*H*_3_), 2.43 (6H, s, C*H*_3_CN), 1.47 (6H, t, ^3^*J*_HH_ = 9.5 Hz, C*H*_3_) ppm. ^13^C{^1^H} NMR (125 MHz, CDCl_3_): *δ*_C_ = 182.1 (*C*O), 160.9 (*C*O), 159.9, 143.0, 140.4, 132.5, 131.7, 125.3, 123.5, 121.7, 120.1, 62.4, 17.3, 14.5, 3.7 ppm. HRMS (ES^+^) found *m*/*z* 767.1328, calcd 767.1331 for [IrC_30_H_30_N_4_O_4_S_2_]^+^. UV/Vis (CH_3_CN): *λ*_max_/nm (*ε*/M^−1^ cm^−1^) = 414 (18 000), 306 (56 300), 254 (33 200). IR (solid): *ν* 2985, 2980, 2374, 2322 (C≡N), 1714 (C=O), 1558, 1379, 1290, 1263, 1055 (B–F), 761, 731 cm^−1^.

#### Synthesis of [Ir(emptz)_2_(L^1^)](BF_4_)

[Ir(emptz)_2_(MeCN)_2_](BF_4_) (0.05 g, 0.06 mmol) and L^1^ (0.02 g, 0.06 mmol) were added to CHCl_3_ (8 mL). The reaction was heated to reflux for 24 hours under a N_2_ atmosphere. Solvent was removed *in vacuo* and the product precipitated from DCM/Et_2_O to give the title compound as a red/orange solid. Yield: 0.0447 g, 0.04 mmol, 68%. ^1^H NMR (400 MHz, CDCl_3_): *δ*_H_ = 9.00 (1H, s), 8.84 (1H, s), 8.23 (1H, app. t, ^3^*J*_HH_ = 5.7 Hz), 7.96 (2H, app. dd, *J*_HH_ = 5.7, 16.3 Hz), 7.72–7.69 (2H, m), 7.22 (1H, d, ^3^*J*_HH_ = 5.5 Hz), 7.10 (2H, dd, *J*_HH_ = 7.4, 12.5 Hz), 6.99 (2H, dd, *J*_HH_ = 7.4, 13.5 Hz), 6.42 (2H, d, ^3^*J*_HH_ = 7.6 Hz), 4.32 (4H, q, ^3^*J*_HH_ = 6.9 Hz, C*H*_2_), 3.39 (2H, q, ^3^*J*_HH_ = 6.7 Hz, C*H*_2_), 2.68 (3H, s, C*H*_3_), 1.86 (3H, s, C*H*_3_), 1.84 (3H, s, C*H*_3_), 1.71 (2H, t, ^3^*J*_HH_ = 7.2 Hz, C*H*_2_), 1.35 (6H, t, ^3^*J*_HH_ = 7.1 Hz, C*H*_3_), 1.28–1.24 (10H, br. m, C*H*_2_), 0.85 (3H, t, ^3^*J*_HH_ = 6.7 Hz, C*H*_3_) ppm. ^13^C{^1^H} NMR (100 MHz, CDCl_3_): *δ*_C_ = 182.4 (*C*O), 182.2 (*C*O), 163.0 (*C*O), 160.4, 160.3, 158.5, 158.4, 157.1, 155.6, 153.7, 150.5, 149.5, 149.3, 149.1, 145.4, 140.0, 133.3, 132.6, 132.5, 129.3, 127.4, 127.1, 126.2, 123.8, 123.7, 121.6, 120.6, 120.5, 62.5, 62.4, 41.1, 32.0, 29.4, 29.1, 27.2, 22.8, 21.5, 15.5, 15.1, 14.3 ppm. HRMS (ES^+^) found *m*/*z* 1010.2933, calcd 1010.2949 for [IrC_46_H_51_N_5_O_5_S_2_]^+^. UV/Vis (CH_3_CN): *λ*_max_/nm (*ε*/M^−1^ cm^−1^) = 434 (6100), 365 (7000), 315 sh. (29 300), 298 (34 100), 273 (34 900), 212 (39 300). IR (solid): *ν* 2960, 2926, 1716 br (C=O), 1541, 1373, 1288, 1257, 1089, 1010 br (B–F), 796, 761 cm^−1^.

#### Synthesis of [Ir(emptz)_2_(L^2^)](BF_4_)

Made similarly to [Ir(emptz)_2_(L^1^)](BF_4_) but using [Ir(emptz)_2_(MeCN)_2_](BF_4_) (0.05 g, 0.06 mmol) and L^2^ (0.02 g, 0.06 mmol) in CHCl_3_ (8 mL) to give the title compound as red/orange solid. Yield: 0.0520 g, 0.05 mmol, 77%. ^1^H NMR (400 MHz, CDCl_3_): *δ*_H_ = 9.03 (1H, s), 8.86 (1H, s), 8.29–8.24 (1H, br. s), 7.98 (1H, dd, *J*_HH_ = 1.6, 5.7 Hz), 7.93 (1H, d, ^3^*J*_HH_ = 5.8 Hz), 7.71 (1H, app. qd, *J*_HH_ = 0.9, 4.0 Hz), 7.67 (1H, d, ^3^*J*_HH_ = 5.6 Hz), 7.21 (1H, dd, *J*_HH_ = 0.7, 5.5 Hz), 7.10 (2H, app. qd, *J*_HH_ = 1.1, 8.6 Hz), 6.99 (2H, app. qd, *J*_HH_ = 1.4, 6.8 Hz), 6.42 (2H, d, ^3^*J*_HH_ = 7.6 Hz), 4.37–4.29 (4H, m, C*H*_2_), 3.49 (2H, q, ^3^*J*_HH_ = 6.8 Hz, C*H*_2_), 2.69 (3H, s, C*H*_3_), 1.86 (3H, s, C*H*_3_), 1.84 (3H, s, C*H*_3_), 1.72 (2H, t, ^3^*J*_HH_ = 7.1 Hz, C*H*_2_), 1.35 (6H, q, ^3^*J*_HH_ = 7.0 Hz, C*H*_3_), 1.27–1.24 (14H, br. m, C*H*_2_), 0.86 (3H, t, ^3^*J*_HH_ = 6.9 Hz, C*H*_3_), ppm. ^13^C{^1^H} NMR (100 MHz, CDCl_3_): *δ*_C_ = 182.4 (*C*O), 182.2 (*C*O), 163.1 (*C*O), 160.4, 158.5, 158.4, 157.1, 155.6, 153.7, 150.5, 149.5, 149.3, 149.1, 148.7, 147.2, 145.5, 140.0, 140.0, 133.3, 132.6, 132.5, 129.3, 127.4, 127.1, 126.2, 126.1, 123.8, 123.7, 121.7, 120.7, 120.5, 62.5, 62.4, 41.1, 32.1, 29.8, 29.5, 29.2, 27.2, 22.8, 21.5, 15.6, 15.1, 14.3 ppm. HRMS (ES^+^) found *m*/*z* 1038.3240, calcd 1038.3261 for [IrC_48_H_55_N_5_O_5_S_2_]^+^. UV/Vis (CH_3_CN): *λ*_max_/nm (*ε*/M^−1^ cm^−1^) = 436 (7100), 368 (9200), 308 (33 000), 269 (27 800), 209 (50 900). IR (solid): *ν* 2941, 2926, 2852, 1716 br (C=O), 1541, 1373, 1288, 1255, 1091, 1026 br (B–F), 798, 761 cm^−1^.

#### Synthesis of [Ir(emptz)_2_(L^3^)](BF_4_)

Made similarly to [Ir(emptz)_2_(L^1^)](BF_4_) but using [Ir(emptz)_2_(MeCN)_2_](BF_4_) (0.05 g, 0.06 mmol) and L^3^ (0.02 g, 0.06 mmol) to give the title compound as red/orange solid. Yield: 0.0160 g, 0.01 mmol, 24%. ^1^H NMR (400 MHz, CDCl_3_): *δ*_H_ = 9.01 (1H, s), 8.84 (1H, s), 8.25 (1H, app. t, ^3^*J*_HH_ = 5.0 Hz), 7.96 (2H, d, ^3^*J*_HH_ = 6.9 Hz), 7.71–7.68 (2H, m), 7.22 (1H, d, ^3^*J*_HH_ = 5.4 Hz), 7.09 (2H, app. q, ^3^*J*_HH_ = 6.1 Hz), 6.98 (2H, app. q, ^3^*J*_HH_ = 6.2 Hz), 6.42 (2H, d, ^3^*J*_HH_ = 7.6 Hz), 4.32 (4H, q, ^3^*J*_HH_ = 6.3 Hz, C*H*_2_), 3.47 (2H, q, ^3^*J*_HH_ = 6.8 Hz, C*H*_2_), 2.67 (3H, s, C*H*_3_), 1.85 (3H, s, C*H*_3_), 1.83 (3H, s, C*H*_3_), 1.71 (2H, t, ^3^*J*_HH_ = 6.9 Hz, C*H*_2_), 1.34 (6H, t, ^3^*J*_HH_ = 7.0 Hz, C*H*_3_), 1.23–1.21 (18H, br. m, C*H*_2_), 0.86 (3H, t, ^3^*J*_HH_ = 6.4 Hz, C*H*_3_) ppm. ^13^C{^1^H} NMR (100 MHz, CDCl_3_): *δ*_C_ = 182.4 (*C*O), 182.2 (*C*O), 162.9 (*C*O), 160.4, 160.3, 158.5, 158.4, 157.1, 155.6, 153.7, 150.5, 149.4, 149.3, 149.1, 145.4, 133.2, 132.5, 132.4, 129.3, 127.3, 127.1, 126.2, 126.1, 123.8, 123.7, 121.6, 120.6, 120.5, 66.0, 62.4, 41.1, 32.0, 29.8, 29.7, 29.5, 29.1, 22.8, 21.4, 15.5, 15.4, 15.1, 14.3, 14.2 ppm. LRMS (ES^+^) found *m*/*z* 1066.35, calcd 1066.36 for [M − BF_4_]^+^; HRMS (ES^+^) found *m*/*z* 1066.3556, calcd 1066.3573 for [IrC_50_H_59_N_5_O_5_S_2_]^+^. UV/Vis (CH_3_CN): *λ*_max_/nm (*ε*/M^−1^ cm^−1^) = 435 (8000), 299 (42 300), 269 (41 600). IR (solid): *ν* 2924, 2852, 1716 (C=O), 1699 (C=O), 1543, 1541, 1456, 1373, 1288, 1257, 1097, 1056 br (B–F), 669 cm^−1^.

#### Synthesis of [Ir(epqc)_2_(L^1^)](BF_4_)

Made similarly to [Ir(emptz)_2_(L^1^)](BF_4_) but using [Ir(epqc)_2_(MeCN)_2_](BF_4_) (0.05 g, 0.05 mmol) and L^1^ (0.02 g, 0.05 mmol) in CHCl_3_ (8 mL) to give the title compound as a red/brown solid. Yield: 0.0503 g, 0.04 mmol, 87%. ^1^H NMR (400 MHz, CDCl_3_): *δ*_H_ = 8.62 (1H, dd, *J*_HH_ = 0.9, 14.0 Hz), 8.61 (1H, s), 8.57 (1H, s), 8.54 (1H, dd, *J*_HH_ = 1.0, 8.5 Hz), 8.41 (1H, s), 8.19 (1H, d, ^3^*J*_HH_ = 5.8 Hz), 8.12 (1H, app. t, ^3^*J*_HH_ = 5.7 Hz), 8.06 (2H, app. t, ^3^*J*_HH_ = 8.2 Hz), 8.01 (1H, dd, *J*_HH_ = 1.6, 5.8 Hz), 7.90 (1H, d, ^3^*J*_HH_ = 5.7 Hz), 7.48–7.36 (4H, m), 7.20 (3H, app. q, *J*_HH_ = 7.6 Hz), 7.09–7.05 (1H, m), 7.02–6.98 (1H, m), 6.87–6.81 (2H, m), 6.50 (2H, dd, *J*_HH_ = 2.7, 7.1 Hz), 4.61 (4H, q, ^3^*J*_HH_ = 7.1 Hz, C*H*_2_), 3.44–3.39 (2H, m, C*H*_2_), 2.52 (3H, s, C*H*_3_), 1.64 (2H, t, ^3^*J*_HH_ = 6.9 Hz, C*H*_2_), 1.55 (6H, app. td, *J*_HH_ = 2.2, 7.2 Hz, C*H*_3_), 1.28–1.21 (10H, br. m, C*H*_2_), 0.83 (3H, t, ^3^*J*_HH_ = 6.8 Hz, C*H*_3_) ppm. ^13^C{^1^H} NMR (125 MHz, CDCl_3_): *δ*_C_ = 169.6 (*C*O), 165.1 (*C*O), 162.6 (*C*O), 156.4, 155.0, 153.4, 151.1, 151.0, 148.3, 148.2, 147.9, 145.1, 144.8, 139.0, 138.9, 134.9, 132.0, 131.7, 131.6, 131.5, 129.1, 128.9, 128.6, 128.3, 127.7, 127.3, 127.2, 126.9, 126.5, 146.4, 63.0, 41.1, 32.0, 29.4, 29.1, 27.2, 22.8, 21.3, 14.5, 14.2, 1.2 ppm. HRMS (ES^+^) found *m*/*z* 1070.3805, calcd 1070.3819 for [IrC_56_H_55_N_5_O_5_]^+^. UV/Vis (CH_3_CN): *λ*_max_/nm (*ε*/M^−1^ cm^−1^) = 461 (4500), 351 (25 400), 289 (63 000), 264 (70 500), 208 (92 300). IR (solid): *ν* 2962, 2924, 1718 br (C=O), 1373, 1539, 1259, 1238, 1078, 1014 br (B–F), 792, 761 cm^−1^.

#### Synthesis of [Ir(epqc)_2_(L^2^)](BF_4_)

Made similarly to [Ir(emptz)_2_(L^1^)](BF_4_) but using [Ir(epqc)_2_(MeCN)_2_](BF_4_) (0.05 g, 0.05 mmol) and L^2^ (0.02 g, 0.05 mmol) in CHCl_3_ (8 mL) to give the title compound as a red/brown solid. Yield: 0.0492 g, 0.04 mmol, 83%. ^1^H NMR (400 MHz, CDCl_3_): *δ*_H_ = 8.63–8.56 (3H, m), 8.56 (1H, s), 8.54 (1H, dd, *J*_HH_ = 1.0, 8.5 Hz), 8.43 (1H, s), 8.17 (1H, d, ^3^*J*_HH_ = 5.6 Hz), 8.11–8.04 (3H, m), 8.01 (1H, dd, *J*_HH_ = 1.6, 5.7 Hz), 7.89 (1H, d, ^3^*J*_HH_ = 5.8 Hz), 7.49–7.43 (2H, m), 7.39 (2H, app. t, ^3^*J*_HH_ = 7.9 Hz), 7.19 (2H, app. t, ^3^*J*_HH_ = 7.3 Hz), 7.09–7.05 (1H, m), 7.02–6.98 (1H, m), 6.87–6.82 (2H, m), 6.50 (2H, d, ^3^*J*_HH_ = 8.0 Hz), 4.62 (4H, q, ^3^*J*_HH_ = 7.1 Hz, C*H*_2_), 3.41 (2H, q, ^3^*J*_HH_ = 6.7 Hz, C*H*_2_), 2.54 (3H, s, C*H*_3_), 1.66–1.62 (2H, m, C*H*_2_), 1.56 (6H, app. td, *J*_HH_ = 2.2, 7.1 Hz, C*H*_3_), 1.29–1.22 (14H, m, C*H*_2_), 0.85 (3H, t, ^3^*J*_HH_ = 6.9 Hz, C*H*_3_) ppm. ^13^C{^1^H} NMR (100 MHz, CDCl_3_): *δ*_C_ = 169.4 (*C*O), 165.1 (*C*O), 162.5 (*C*O), 156.4, 155.0, 153.4, 148.2, 147.9, 145.2, 145.1, 138.9, 132.0, 131.5, 128.9, 128.6, 127.1, 126.9, 125.2, 124.6, 123.6, 121.2, 118.9, 118.5, 63.0, 32.0, 29.7, 27.2, 22.8, 21.3, 14.5, 14.3 ppm. HRMS (ES^+^) found *m*/*z* 1098.4111, calcd 1098.4131 for [IrC_58_H_59_N_5_O_5_]^+^. UV/Vis (CH_3_CN): *λ*_max_/nm (*ε*/M^−1^ cm^−1^) = 461 (2600), 355 (14 000), 289 (25 900), 267 (29 900), 247 (26 600), 210 (47 700). IR (solid): *ν* 2961, 2926, 2853, 1719 br (C=O), 1375, 1539, 1261, 1240, 1065, 1016 br (B–F), 760, 762 cm^−1^.

#### Synthesis of [Ir(epqc)_2_(L^3^)](BF_4_)

Made similarly to [Ir(emptz)_2_(L^1^)](BF_4_) but using [Ir(epqc)_2_(MeCN)_2_](BF_4_) (0.05 g, 0.05 mmol) and L^3^ (0.02 g, 0.05 mmol) in CHCl_3_ (8 mL) to give the title compound as a red/brown solid. Yield: 0.0485 g, 0.04 mmol, 73%. ^1^H NMR (400 MHz, CDCl_3_): *δ*_H_ = 8.61 (1H, d, ^3^*J*_HH_ = 15.3 Hz), 8.61 (1H, s), 8.56 (1H, s), 8.53 (1H, d, ^3^*J*_HH_ = 8.4 Hz), 8.41 (1H, s), 8.18 (1H, d, ^3^*J*_HH_ = 5.8 Hz), 8.14–8.10 (1H, m), 8.06 (2H, app. t, ^3^*J*_HH_ = 8.5 Hz), 8.00 (1H, d, ^3^*J*_HH_ = 5.3 Hz), 7.90 (1H, d, ^3^*J*_HH_ = 5.5 Hz), 7.45–7.38 (4H, m), 7.22–7.17 (3H, m), 7.07 (1H, app. t, ^3^*J*_HH_ = 7.5 Hz), 7.00 (1H, app. t, ^3^*J*_HH_ = 7.8 Hz), 6.84 (2H, app. q, ^3^*J*_HH_ = 6.5 Hz), 6.50 (2H, d, ^3^*J*_HH_ = 7.0 Hz), 4.60 (4H, q, ^3^*J*_HH_ = 6.9 Hz, C*H*_2_), 3.41 (2H, q, ^3^*J*_HH_ = 6.8 Hz, C*H*_2_), 2.52 (3H, s, C*H*_3_), 1.65–1.62 (2H, m, C*H*_2_), 1.55 (6H, t, ^3^*J*_HH_ = 6.6 Hz, C*H*_3_), 1.28–1.19 (18H, m, C*H*_2_), 0.86 (3H, t, ^3^*J*_HH_ = 6.3 Hz, C*H*_3_) ppm. ^13^C{^1^H} NMR (100 MHz, CDCl_3_): *δ*_C_ = 169.5 (*C*O), 165.2 (*C*O), 165.0 (*C*O), 156.4, 155.0, 153.4, 151.1, 151.0, 148.3, 148.2, 147.9, 146.4, 145.2, 145.1, 144.7, 138.9, 138.8, 134.9, 134.8, 131.9, 131.6, 131.5, 131.4, 128.8, 128.6, 128.3, 127.6, 127.3, 127.1, 126.9, 126.4, 125.2, 125.1, 124.6, 124.4, 123.6, 123.4, 121.2, 118.9, 118.5, 66.0, 63.0, 62.9, 41.0, 32.0, 29.8, 29.7, 29.5, 29.4, 29.1, 27.2, 22.8, 21.3, 15.4, 14.5, 14.2 ppm. LRMS (ES^+^) found *m*/*z* 1126.45, calcd 1126.45 for [M − BF_4_]^+^; HRMS (ES^+^) found *m*/*z* 1126.4438, calcd 1126.4443 for [IrC_60_H_63_N_5_O_5_]^+^. UV/Vis (CH_3_CN): *λ*_max_/nm (*ε*/M^−1^ cm^−1^) = 468 (2700), 354 (15 300), 354 (15 300), 288 (28 900), 262 (31 800), 247 (29 600), 211 (53 400). IR (solid): *ν* 2961, 2922, 1719 br (C=O), 1539, 1375, 1256, 1240, 1078, 1013 br (B–F), 791, 764 cm^−1^.

#### Synthesis of [Ir(mptca)_2_(L^3^)]Cl

[Ir(emptz)_2_(L^3^)](BF_4_) (0.03 g, 0.03 mmol) and KOH (1 M, 10 mL) in acetone (10 mL) were stirred at RT for 24 hours under a N_2_ atmosphere. Solvent was removed *in vacuo*, water (approx. 20 mL) was added and the solution neutralised with HCl (1 M). Water was removed *in vacuo* and the crude product dissolved in MeOH (10 mL). The solution was filtered to remove salts and dried *in vacuo* to give the title compound as an orange solid. Yield: 0.0243 g, 0.02 mmol, 85%. ^1^H NMR (400 MHz, CD_3_OD): *δ*_H_ = 8.98 (1H, s), 8.61 (1H, s), 8.08 (1H, d, ^3^*J*_HH_ = 5.8 Hz), 7.81 (2H, app. t, ^3^*J*_HH_ = 4.5 Hz), 7.70 (2H, d, ^3^*J*_HH_ = 7.7 Hz), 7.67 (1H, d, ^3^*J*_HH_ = 5.5 Hz), 7.02 (2H, app. t, ^3^*J*_HH_ = 7.1 Hz), 6.91 (2H, app. t, ^3^*J*_HH_ = 7.5 Hz), 6.44 (2H, app. t, ^3^*J*_HH_ = 7.4 Hz), 3.38 (2H, t, ^3^*J*_HH_ = 7.1 Hz, C*H*_2_), 2.57 (3H, s, C*H*_3_), 1.81 (6H, s, C*H*_3_), 1.64–1.55 (2H, m, C*H*_2_), 1.29–1.24 (18H, m, C*H*_2_), 0.85 (3H, t, ^3^*J*_HH_ = 6.3 Hz, C*H*_3_) ppm. ^13^C{^1^H} NMR (100 MHz, CD_3_OD): *δ*_C_ = 180.8 (*C*O), 166.7 (*C*O), 165.9 (*C*O), 158.9, 157.1, 154.5, 153.8, 152.1, 151.0, 149.9, 149.8, 146.0, 142.3, 142.2, 134.3, 134.1, 132.2, 130.5, 126.8, 126.3, 124.2, 123.2, 41.5, 30.8, 30.7, 30.5, 30.4, 30.2, 28.2, 21.4, 20.9, 15.2, 15.0, 14.4 ppm. LRMS (ES^+^) found *m*/*z* 1010.30, calcd 1010.30 for [M − Cl]^+^; HRMS (ES^+^) found *m*/*z* 1010.2956, calcd 1010.2961 for [IrC_46_H_51_N_5_O_5_S_2_]^+^. UV/Vis (CH_3_CN): *λ*_max_/nm (*ε*/M^−1^ cm^−1^) = 417 (2000), 311 (4100), 281 (5300), 251 (9900), 216 (37 900). IR (solid): *ν* 3289 br (O–H), 2922, 2851, 1653 br (C=O), 1541, 1437, 1350, 1277, 1238, 1026, 754, 739 cm^−1^.

#### Synthesis of [Ir(pqca)_2_(L^3^)]Cl

Made as for [Ir(mptca)_2_(L^3^)]Cl but using [Ir(epqc)_2_(L^3^)](BF_4_) (0.02 g, 0.02 mmol) and KOH (1 M, 10 mL) in acetone (10 mL) to give the title compound as an orange solid. Yield: 0.0183 g, 0.02 mmol, 96%. ^1^H NMR (400 MHz, CD_3_OD): *δ*_H_ = 8.61 (1H, s), 8.37–8.35 (3H, m), 8.26 (1H, s), 8.20 (2H, d, ^3^*J*_HH_ = 8.6 Hz), 8.16 (2H, d, ^3^*J*_HH_ = 9.4 Hz), 8.10 (1H, d, ^3^*J*_HH_ = 5.7 Hz), 7.88–7.83 (1H, m), 7.43–7.38 (3H, m), 7.34 (2H, app. t, ^3^*J*_HH_ = 7.6 Hz), 7.18–7.14 (2H, m), 7.02–6.96 (2H, m), 6.79 (2H, app. td, ^3^*J*_HH_ = 2.7, 7.4 Hz), 6.54–6.51 (2H, m), 3.61–3.60 (2H, m, C*H*_2_), 2.47 (3H, s, C*H*_3_), 1.59–1.56 (2H, m, C*H*_2_), 1.34–1.27 (18H, br. m, C*H*_2_), 0.89 (3H, t, ^3^*J*_HH_ = 6.7 Hz, C*H*_3_) ppm. ^13^C{^1^H} NMR (100 MHz, CD_3_OD): *δ*_C_ = 170.6 (*C*O), 170.4 (*C*O), 165.6 (*C*O), 158.0, 157.9, 156.2, 153.6, 151.8, 149.6, 149.2, 149.1, 148.5, 147.5, 147.4, 147.3, 145.7, 135.7, 135.6, 131.6, 131.5, 129.0, 128.3, 128.0, 127.6, 126.2, 124.1, 124.0, 122.5, 115.8, 115.7, 73.8, 71.7, 70.8, 64.4, 57.0, 55.1, 41.4, 30.0, 30.8, 30.7, 30.6, 30.5, 29.9, 28.1, 24.2, 23.7, 22.0, 21.7, 21.2, 14.4 ppm. LRMS (ES^+^) found *m*/*z* 1070.39, calcd 1070.38 for [M − Cl]^+^; HRMS (ES^+^) found *m*/*z* 1070.3835, calcd 1070.3831 for [IrC_56_H_55_N_5_O_5_]^+^. UV/Vis (CH_3_CN): *λ*_max_/nm (*ε*/M^−1^ cm^−1^) = 457 (700), 355 (1600), 288 (3000), 239 (6800). IR (solid): *ν* 3391 br (O–H), 2970, 2926, 1589 br (C=O), 1379, 1375, 1339, 1152, 768, 662 cm^−1^.

## Conflicts of interest

There are no conflicts to declare.

## Supplementary Material

RA-014-D3RA06764E-s001

RA-014-D3RA06764E-s002
