# Supplementary material for: Alkyl chain functionalised Ir(iii) complexes: synthesis, properties and behaviour as emissive dopants in microemulsions
Source: RSC Adv. 2024 Feb 27;14(10):6987–97. doi: 10.1039/d3ra06764e (PMC10897649; doi:10.1039/d3ra06764e)
Supplement: RA-014-D3RA06764E-s001 [file RA-014-D3RA06764E-s001.pdf]

Electronic Supplementary Material (ESI) for RSC Advances.
This journal is © The Royal Society of Chemistry 2024

Alkyl chain functionalised Ir(III) complexes: synthesis, properties and behaviour as emissive dopants in microemulsions

Emily C. Stokes,[a] Ibrahim O. Shoetan,[a] Alice M. Gillman,[a] Peter N. Horton,[b] Simon J. Coles,[b] Simon E. Woodbury,[c] Ian A. Fallis,[a*] and Simon J.A. Pope[a*]

[a] School of Chemistry, Main Building, Park Place, Cardiff University, Cardiff, U.K CF10 3AT

[b] UK National Crystallographic Service, Chemistry, University of Southampton, Highfield, Southampton, UK SO17 1BJ.

[c] National Nuclear Laboratory, Central Laboratory, Sellafield, Seascale, Cumbria CA20 1PG, UK

Electronic Supplementary Information

Contents

Figure S1	^{1}H NMR spectrum of L^1	3
Figure S2	$^{13}C\{^{1}H\}$ NMR spectrum of L^1.	3
Figure S3	^{1}H NMR spectrum of L^2	4
Figure S4	$^{13}C\{^{1}H\}$ NMR spectrum of L^2.	4
Figure S5	^{1}H NMR spectrum of L^3	5
Figure S6	$^{13}C\{^{1}H\}$ NMR spectrum of L^3	5
Figure S7	HRMS data for the ligands, L^1-L^3	6
Figure S8	FT-IR spectra (wavenumber cm^{-1} *vs* transmission %) for the ligands, L^{1-3}	7
Figure S9	^{1}H NMR spectrum of $[Ir(epqc)_2(L^1)]BF_4$	8
Figure S10	$^{13}C\{^{1}H\}$ NMR spectrum of $[Ir(epqc)_2(L^1)]BF_4$	8
Figure S11	^{1}H NMR spectrum of $[Ir(epqc)_2(L^2)]BF_4$	9
Figure S12	$^{13}C\{^{1}H\}$ NMR spectrum of $[Ir(epqc)_2(L^2)]BF_4$	9
Figure S13	^{1}H NMR spectrum of $[Ir(epqc)_2(L^3)]BF_4$	10
Figure S14	$^{13}C\{^{1}H\}$ NMR spectrum of $[Ir(epqc)_2(L^3)]BF_4$	10
Figure S15	^{1}H NMR spectrum of $[Ir(emptz)_2(L^1)]BF_4$	11
Figure S16	$^{13}C\{^{1}H\}$ NMR spectrum of $[Ir(emptz)_2(L^1)]BF_4$	11
Figure S17	^{1}H NMR spectrum of $[Ir(emptz)_2(L^2)]BF_4$	12
Figure S18	$^{13}C\{^{1}H\}$ NMR spectrum of $[Ir(emptz)_2(L^2)]BF_4$	12
Figure S19	^{1}H NMR spectrum of $[Ir(emptz)_2(L^3)]BF_4$	13
Figure S20	$^{13}C\{^{1}H\}$ NMR spectrum of $[Ir(emptz)_2(L^3)]BF_4$	13
Figure S21	HRMS data for the complexes	14
Figure S22	HRMS data for the complexes	15
Figure S23	FT-IR spectra for the complexes	16
Figure S24	FT-IR spectra for the complexes	17
Figure S25	FT-IR spectra for $[Ir(pqca)(L^3)]Cl$ and $[Ir(mptca)(L^3)]Cl$	18
Figure S26	^{1}H NMR spectrum of $[Ir(mptca)_2(L^3)]Cl$ (in CD_3OD).	18
Figure S27	$^{13}C\{^{1}H\}$ NMR spectrum of $[Ir(mptca)_2(L^3)]Cl$ (in CD_3OD).	19
Figure S28	^{1}H NMR spectrum of $[Ir(pqca)_2(L^3)]Cl$ (in CD_3OD).	19
Figure S29	$^{13}C\{^{1}H\}$ NMR spectrum of $[Ir(pqca)_2(L^3)]Cl$ (in CD_3OD	20
Figure S30	HRMS data for $[Ir(pqca)_2(L^3)]Cl$ (top) and $[Ir(mptca)_2(L^3)]Cl$	21

Figure S31	A comparison of the absorption spectra for free epqcH and [Ir(epqc)$_2$(L$^{1-3}$)]BF_4 complexes.	24
Figure S32	A comparison of the absorption spectra for free emptzH and [Ir(emptz)$_2$(L$^{1-3}$)]BF_4 complexes.	24
Figure S33	A comparison of the room and low temperature emission spectra for [Ir(epqc)$_2$(L$^{1-3}$)]BF_4 complexes.	25
Figure S34	A comparison of the room and low temperature emission spectra for [Ir(emptz)$_2$(L$^{1-3}$)]BF_4 complexes.	25

Table S1	Data collection parameters for the X-ray crystal structures.	22
Table S2	Bond lengths and bond angles for the X-ray structures.	23

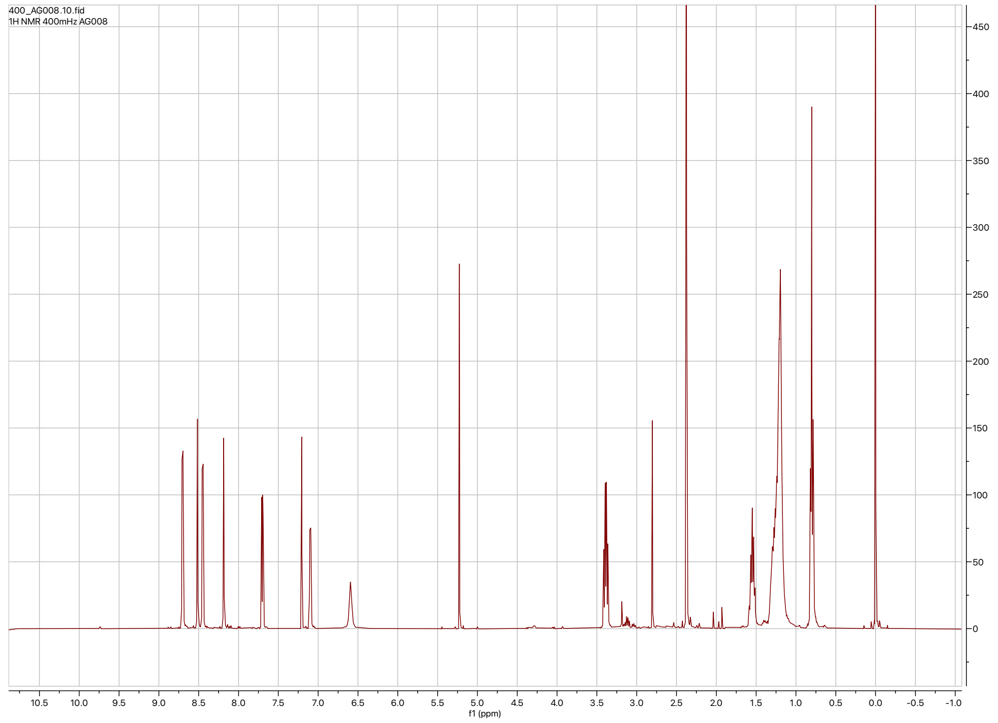

Figure S1. ^{1}H NMR spectrum of L^{1}.

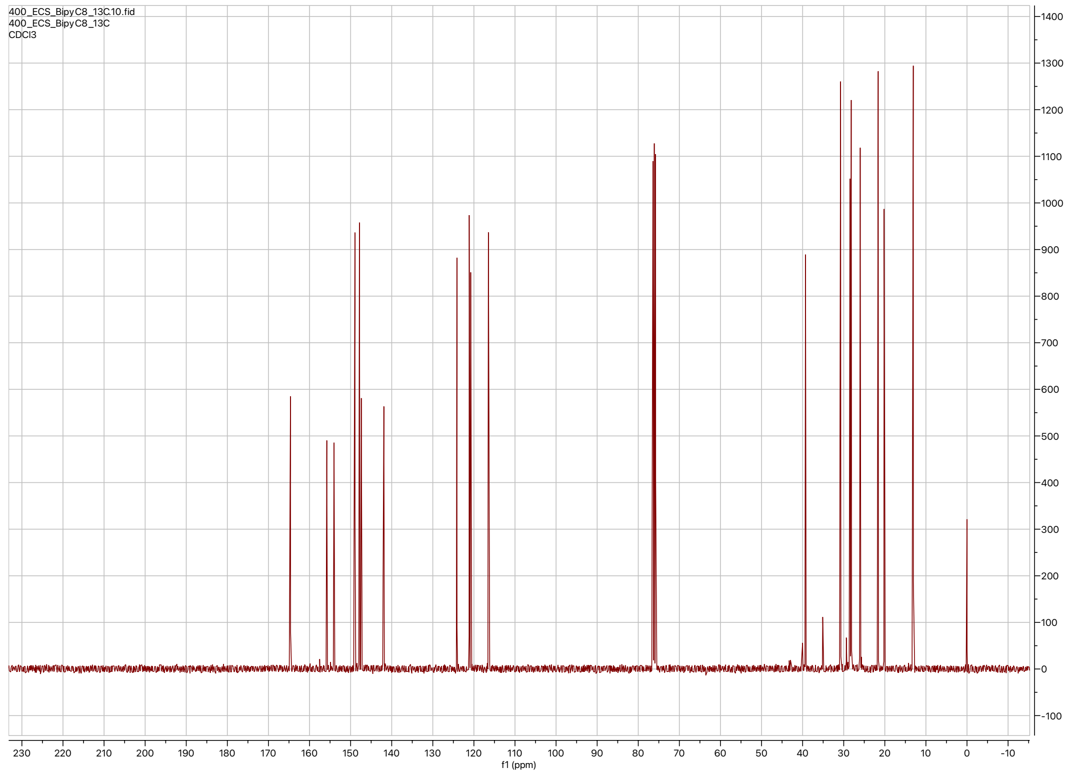

Figure S2. ^{13}C{^{1}H} NMR spectrum of L^{1}.

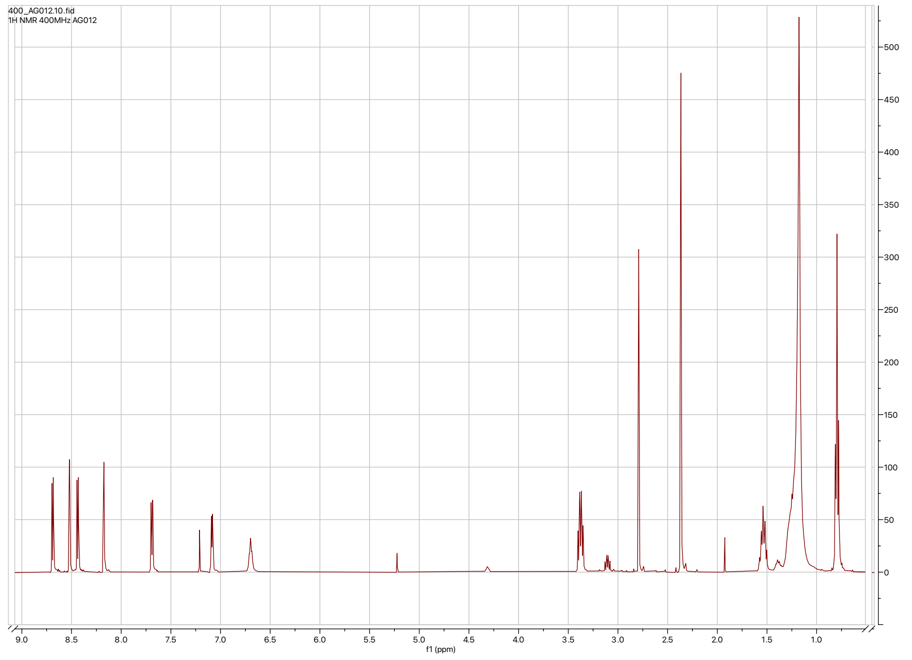

Figure S3. ^{1}H NMR spectrum of L^2.

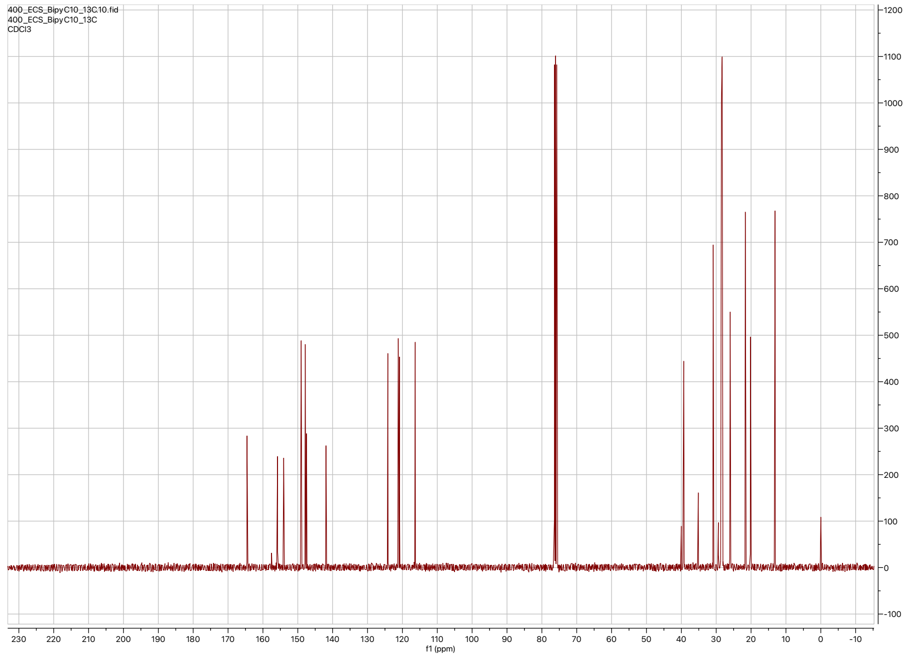

Figure S4. $^{13}C\{^{1}H\}$ NMR spectrum of L^2.

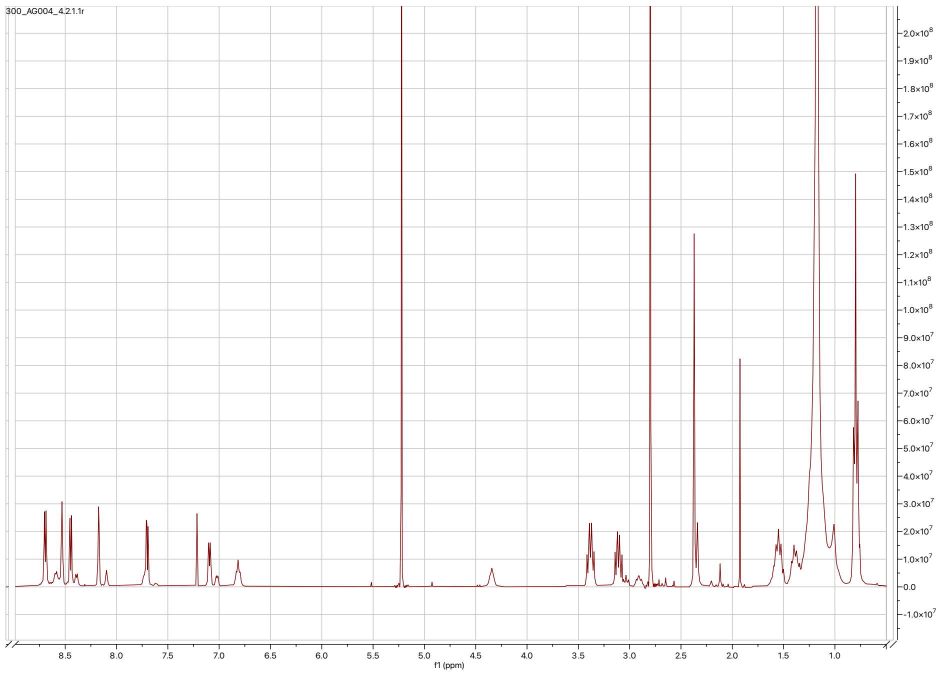

Figure S5. ^{1}H NMR spectrum of L^{3}.

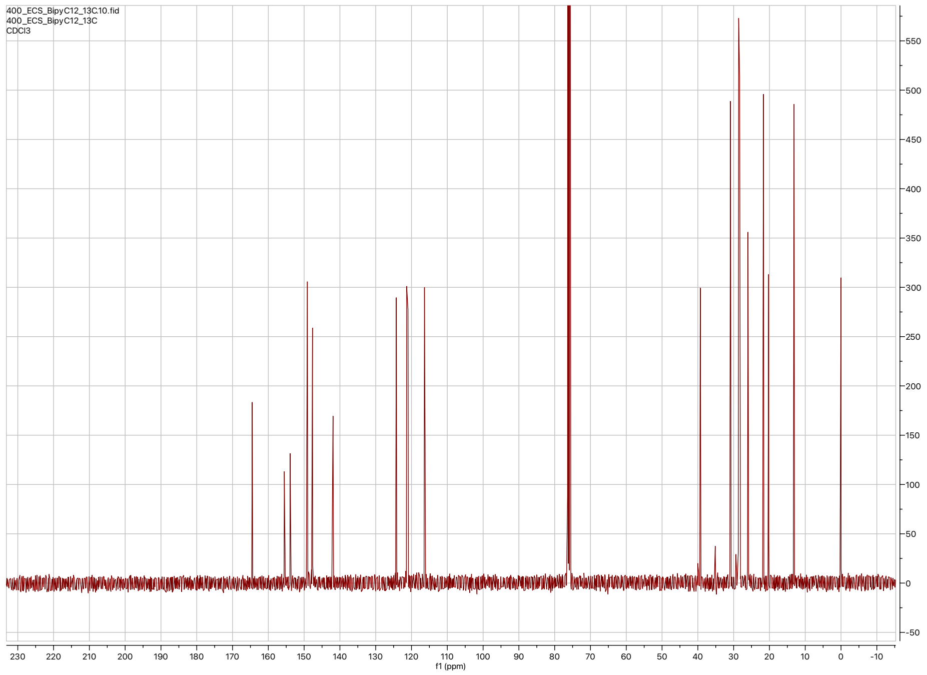

Figure S6. ^{13}C{^{1}H} NMR spectrum of L^{3}.

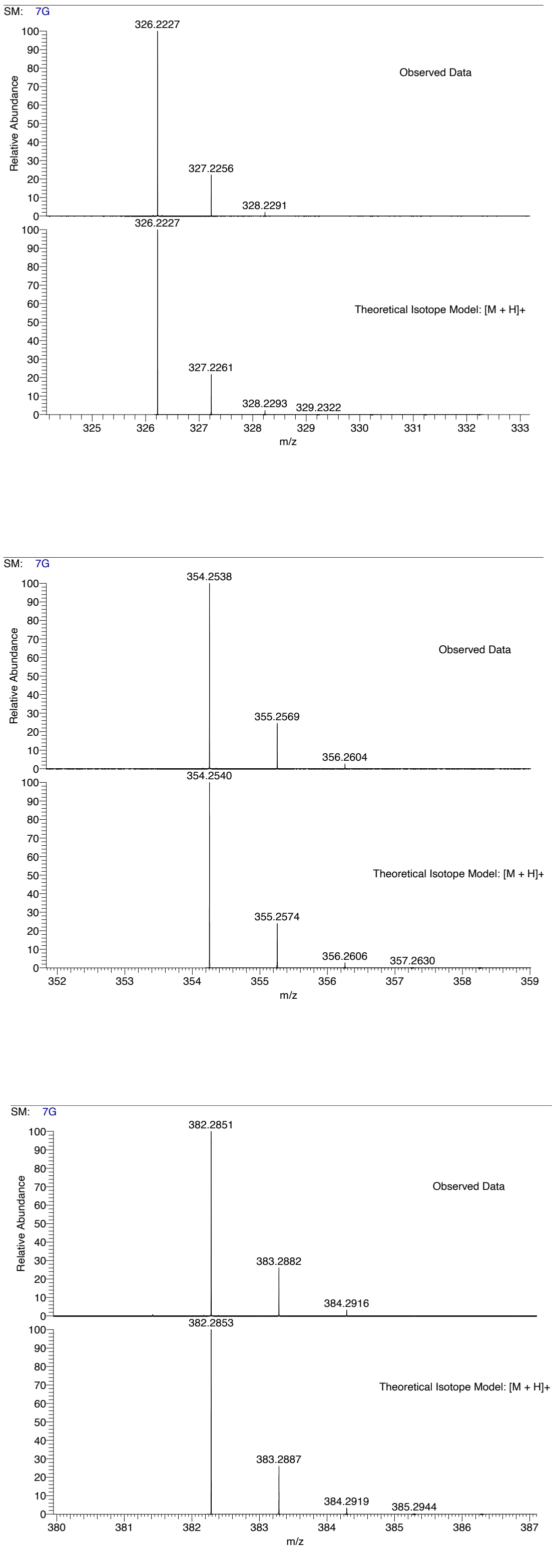

Figure S7. HRMS data for the ligands, L^1-L^3 (top to bottom).

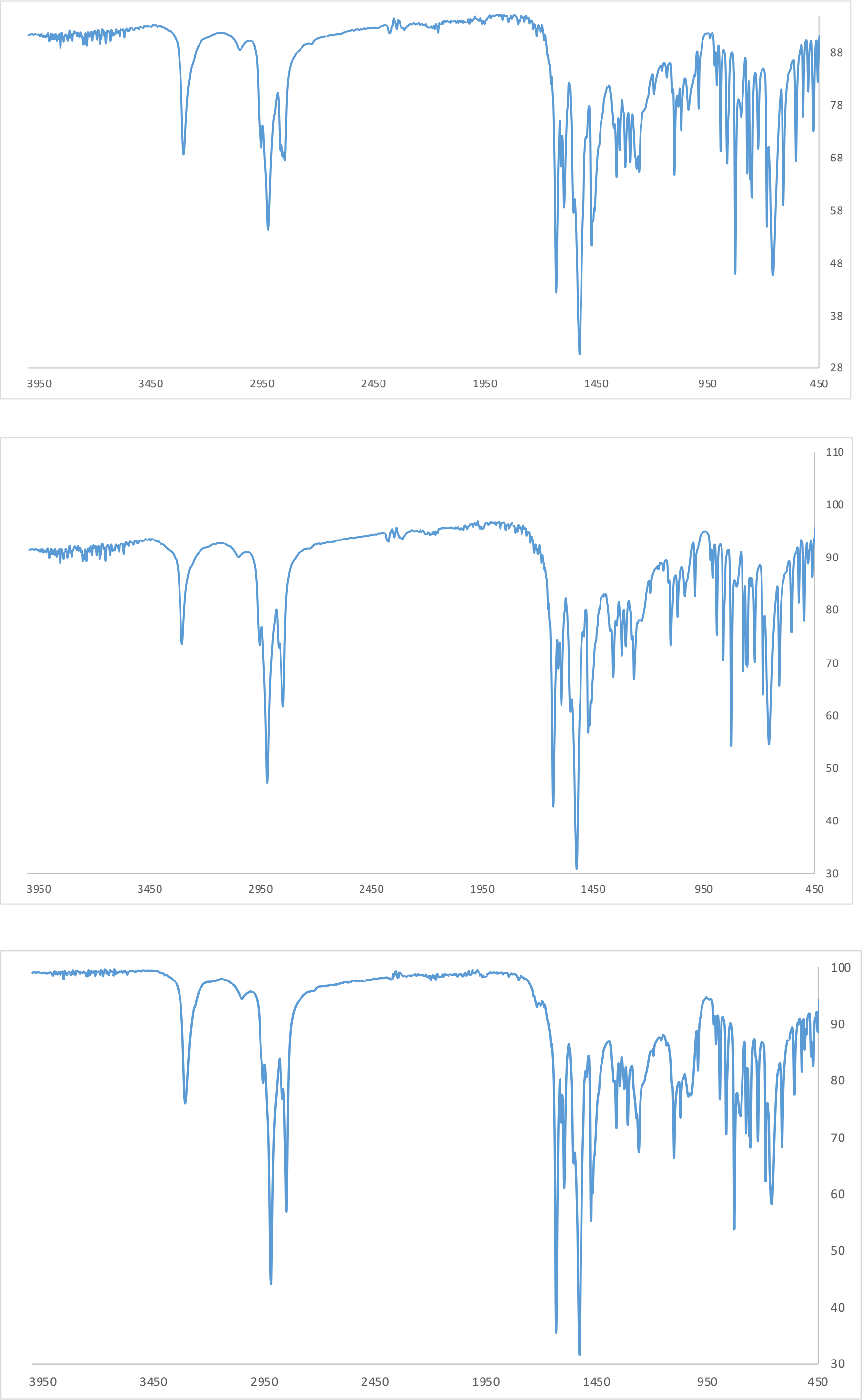

Figure S8. FT-IR spectra (wavenumber cm^{-1} *vs* transmission %) for the ligands, L^{1-3} (top to bottom).

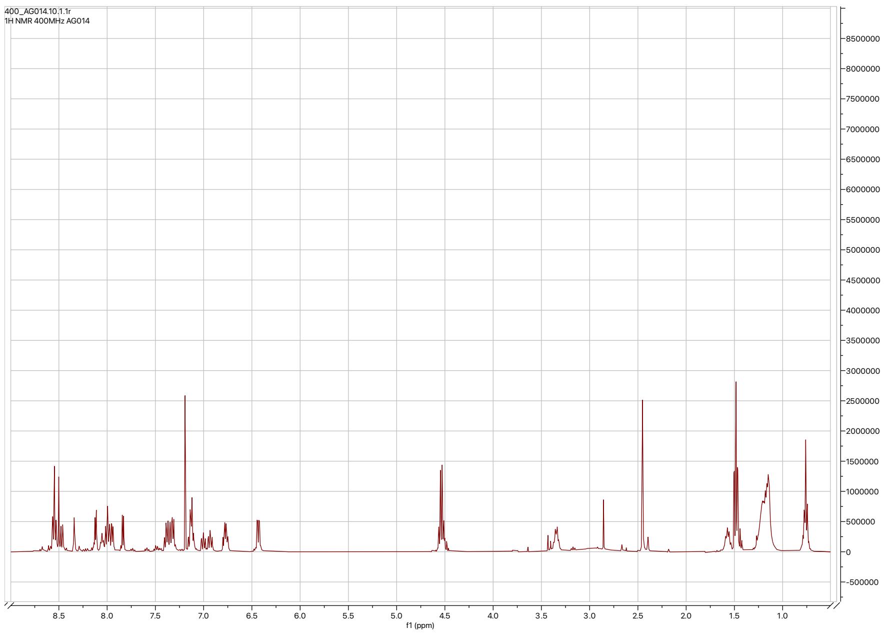

Figure S9. ^{1}H NMR spectrum of $[Ir(epqc)_2(L^1)]BF_4$.

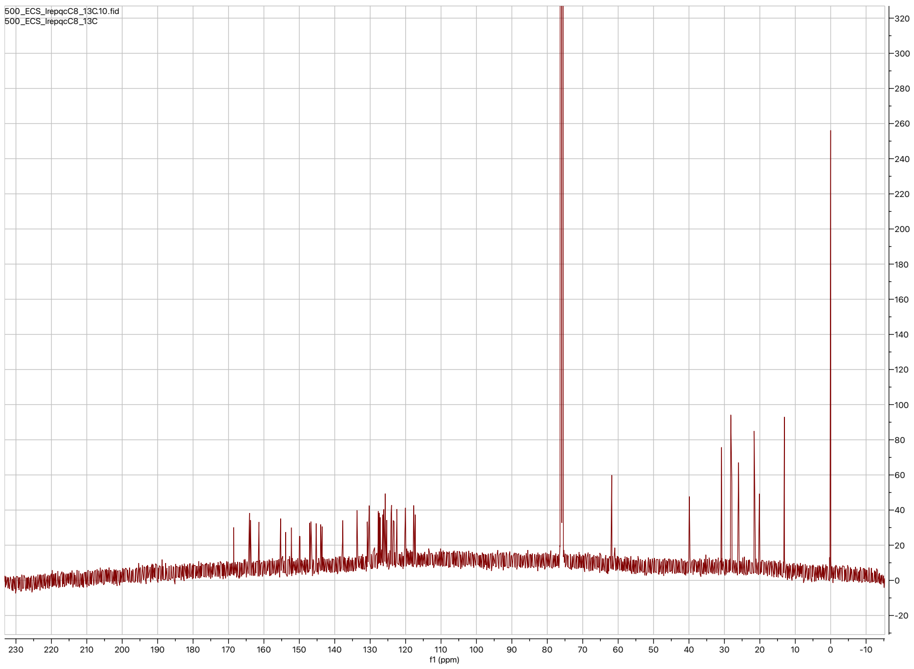

Figure S10. $^{13}C\{^{1}H\}$ NMR spectrum of $[Ir(epqc)_2(L^1)]BF_4$.

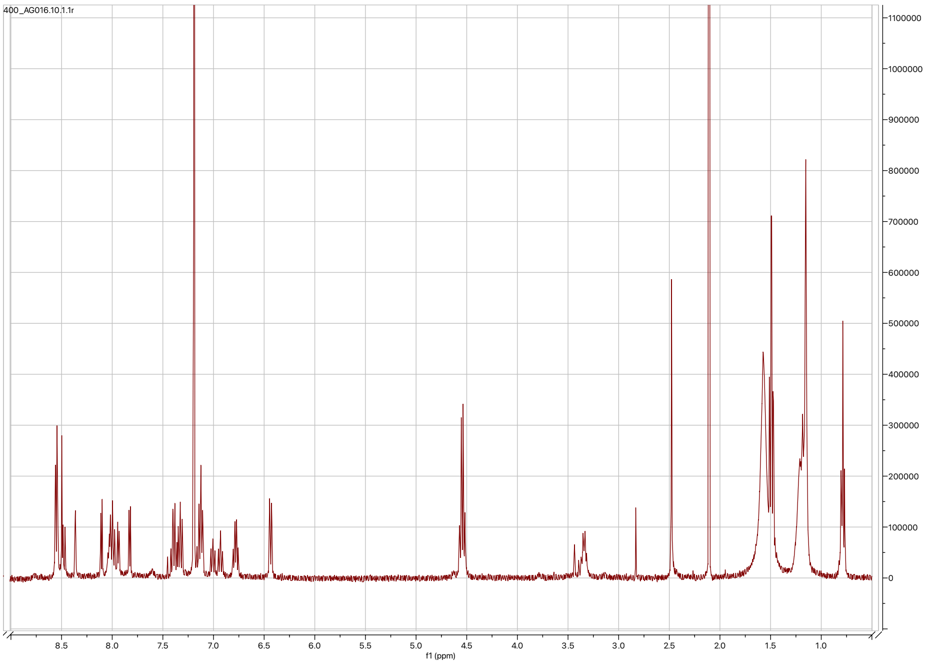

Figure S11. ^{1}H NMR spectrum of $[Ir(epqc)_2(L^2)]BF_4$.

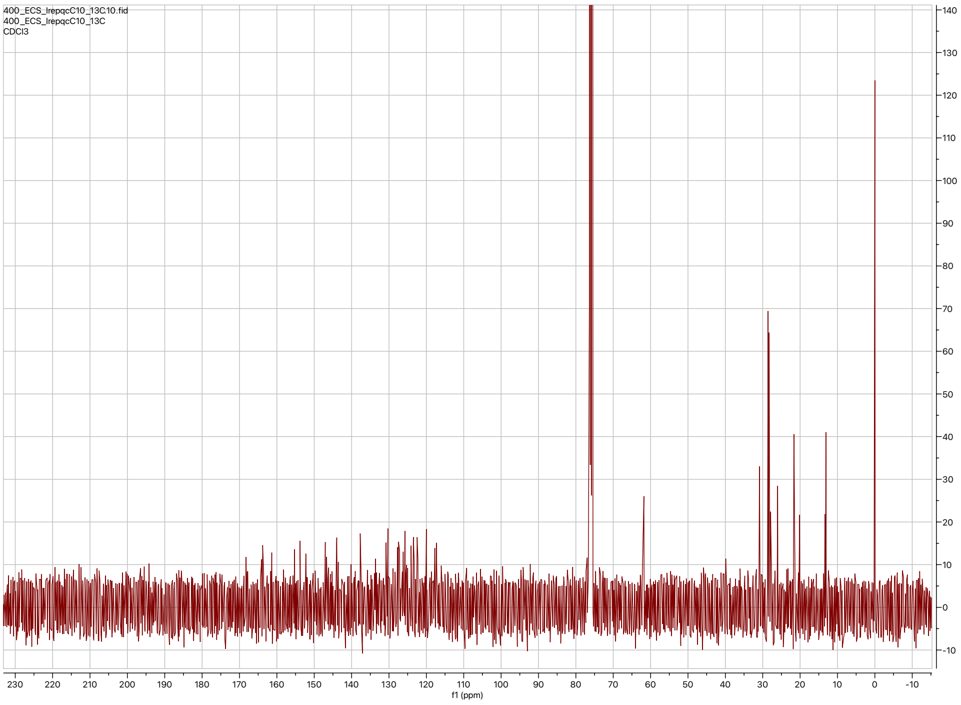

Figure S12. $^{13}C\{^{1}H\}$ NMR spectrum of $[Ir(epqc)_2(L^2)]BF_4$.

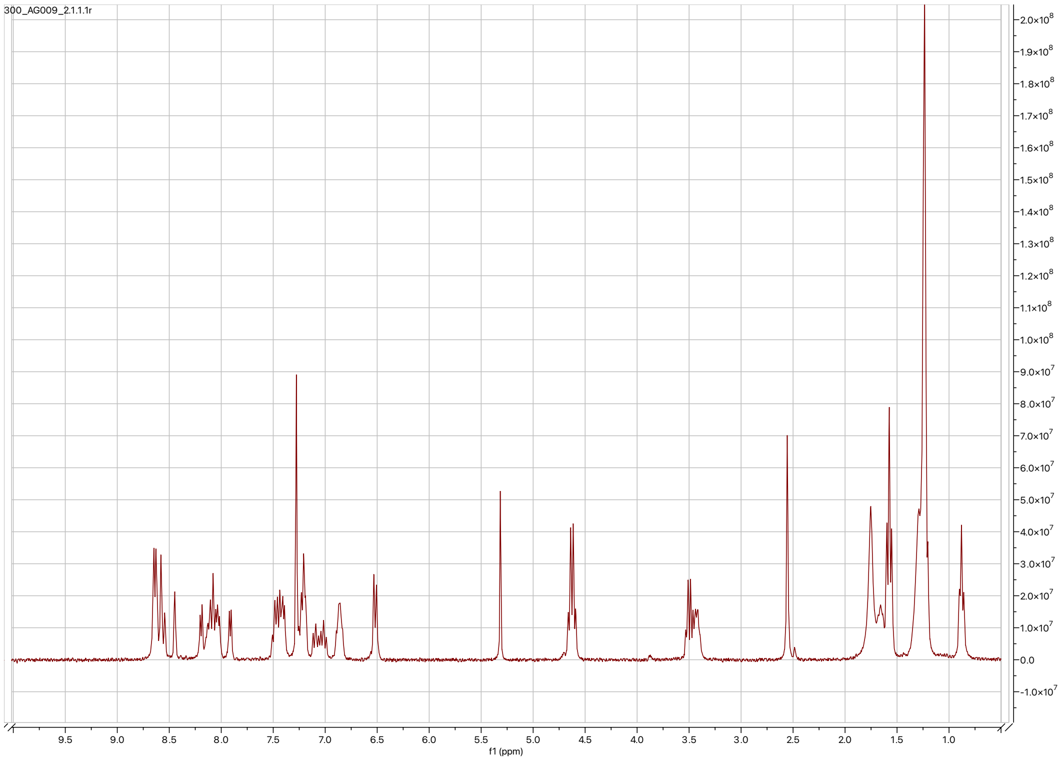

Figure S13. ^{1}H NMR spectrum of $[Ir(epqc)_2(L^3)]BF_4$.

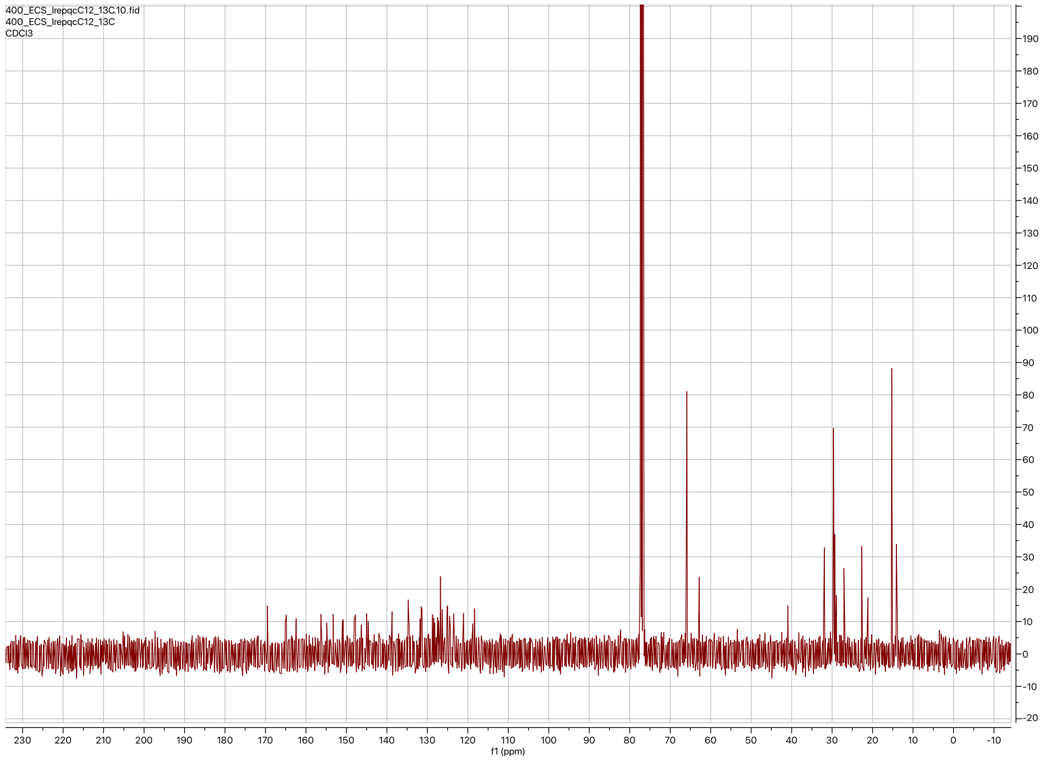

Figure S14. $^{13}C\{^{1}H\}$ NMR spectrum of $[Ir(epqc)_2(L^3)]BF_4$.

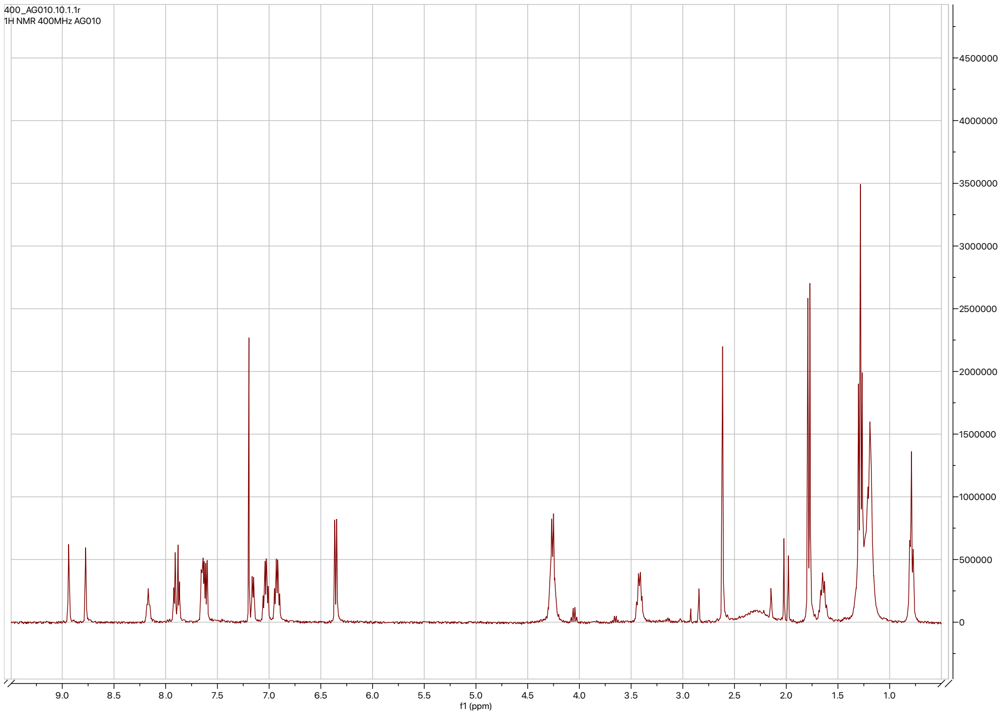

Figure S15. ^{1}H NMR spectrum of $[Ir(emptz)_2(L^1)]BF_4$.

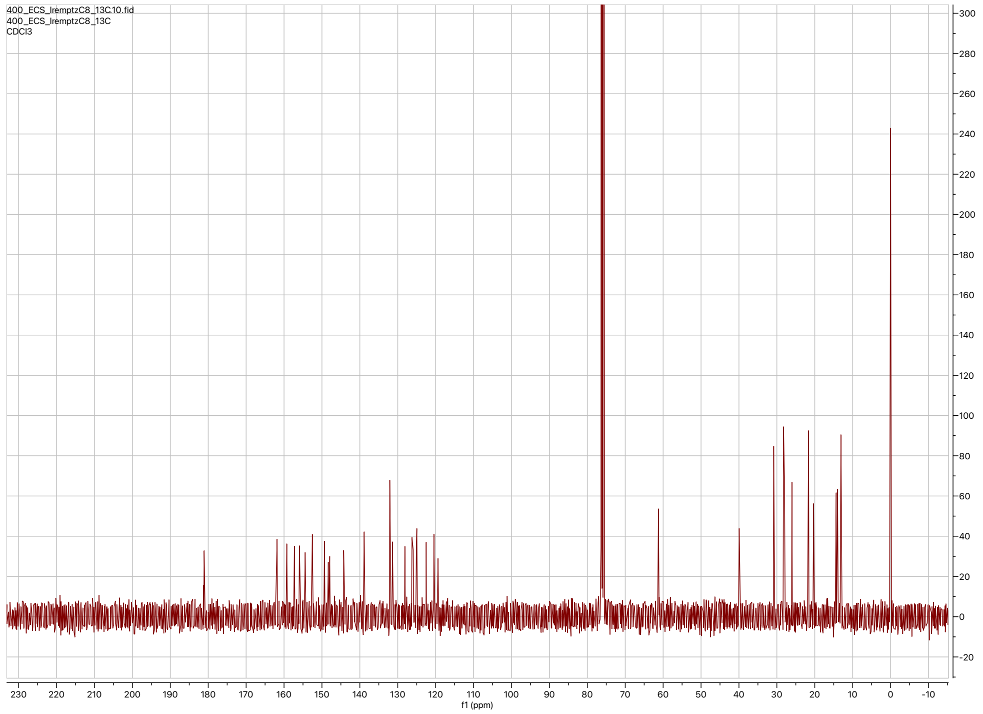

Figure S16. $^{13}C\{^{1}H\}$ NMR spectrum of $[Ir(emptz)_2(L^1)]BF_4$.

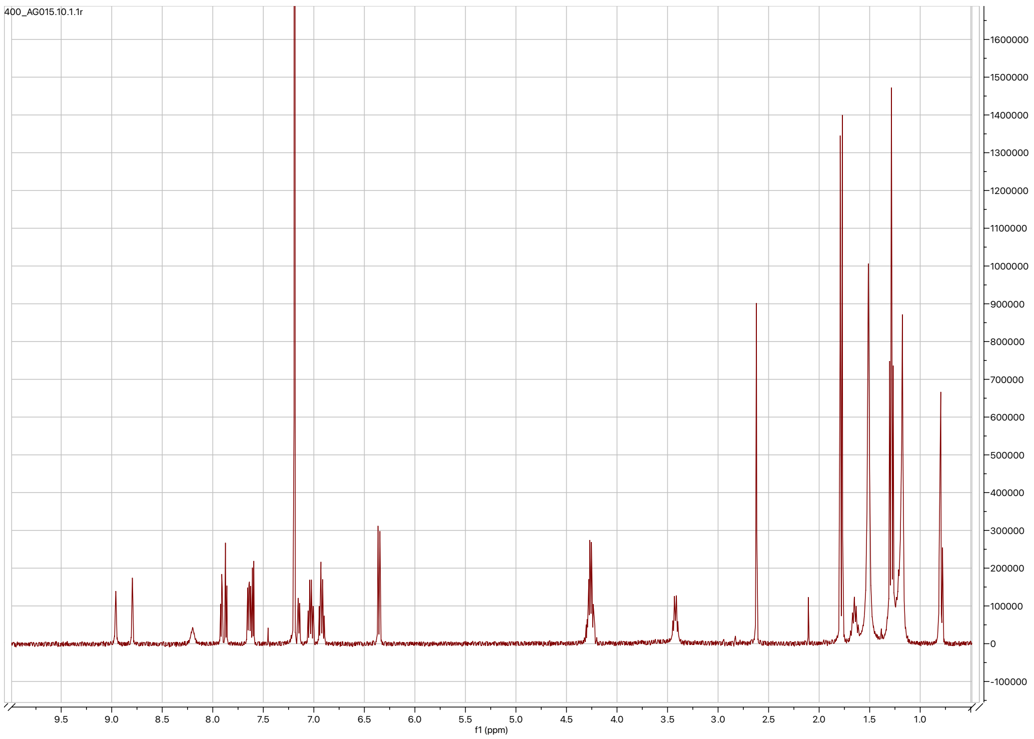

Figure S17. ^{1}H NMR spectrum of $[Ir(emptz)_2(L^2)]BF_4$.

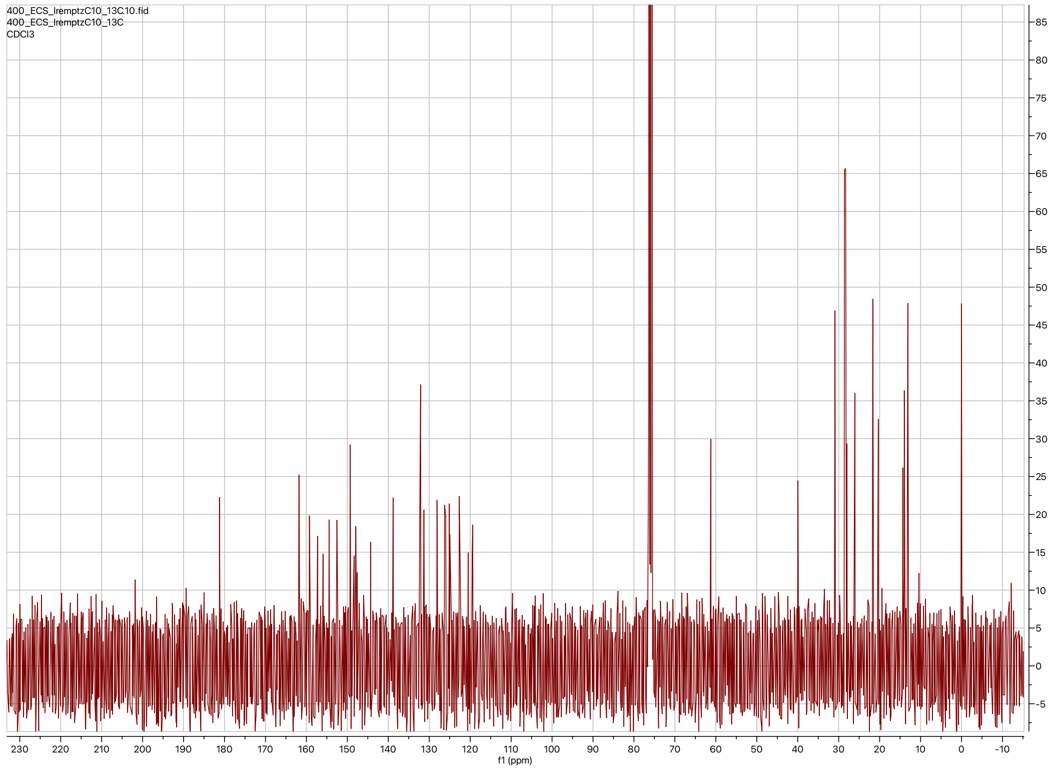

Figure S18. $^{13}C\{^{1}H\}$ NMR spectrum of $[Ir(emptz)_2(L^2)]BF_4$.

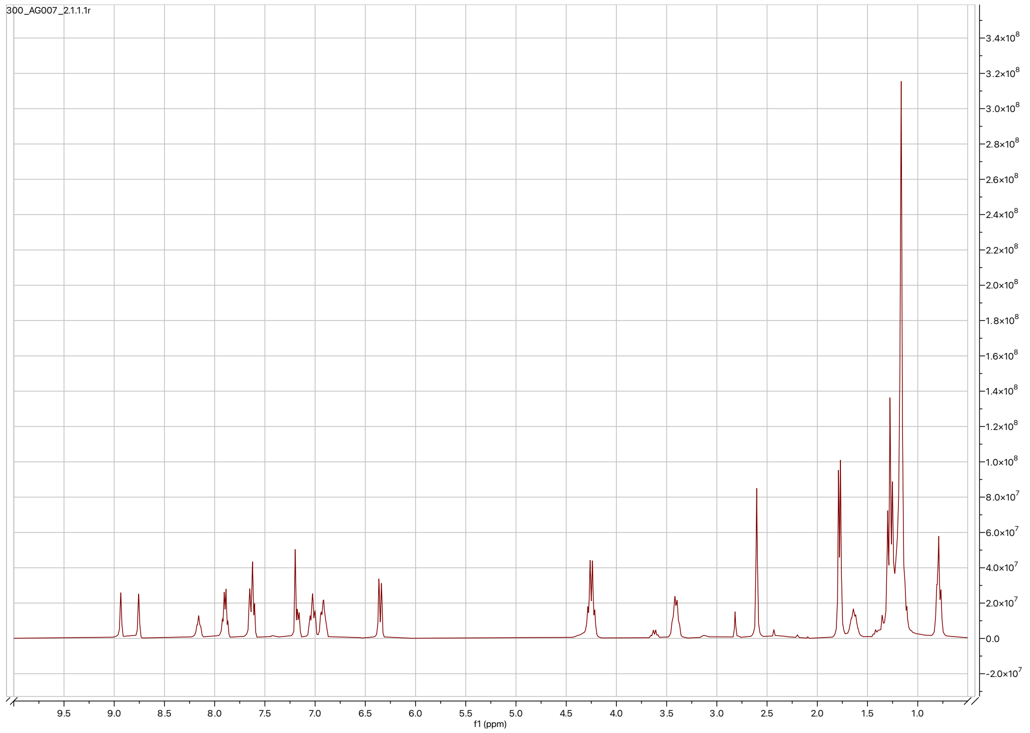

Figure S19. ^{1}H NMR spectrum of $[Ir(emptz)_2(L^3)]BF_4$.

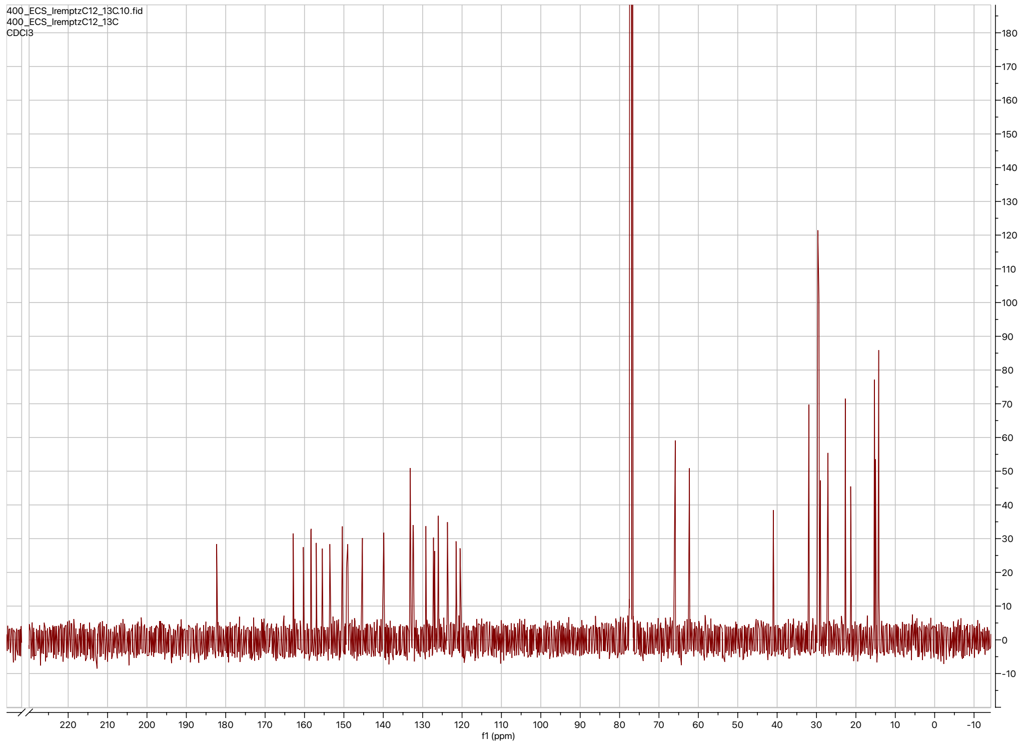

Figure S20. $^{13}C\{^1H\}$ NMR spectrum of $[Ir(emptz)_2(L^3)]BF_4$.

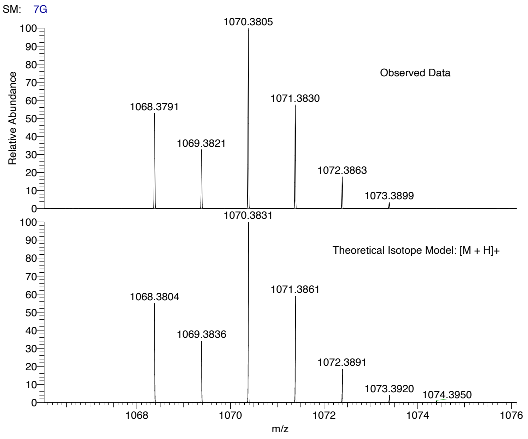

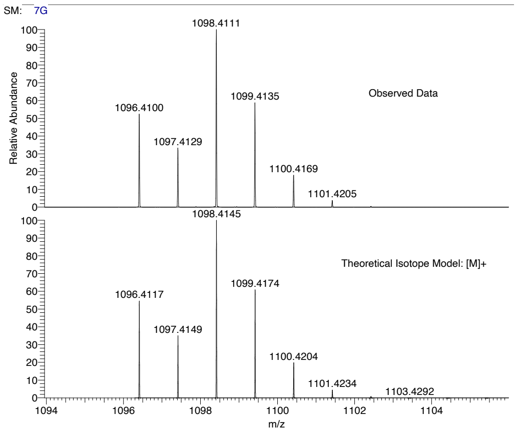

Figure S21. HRMS data for the complexes, [Ir(epqc)(L^{1-2})]BF_4 (top to bottom).

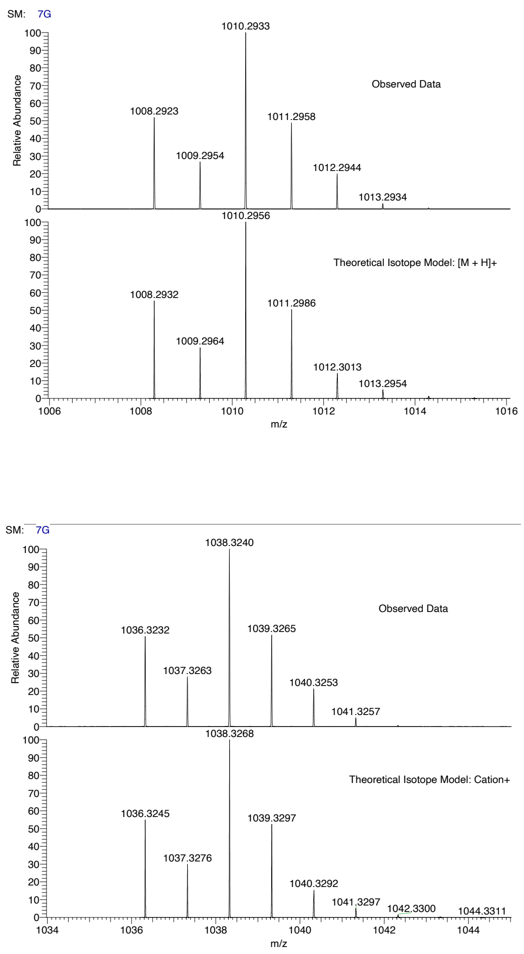

Figure 22. HRMS data for the complexes, [Ir(emptz)(L^{1-2})]BF_4 (top to bottom).

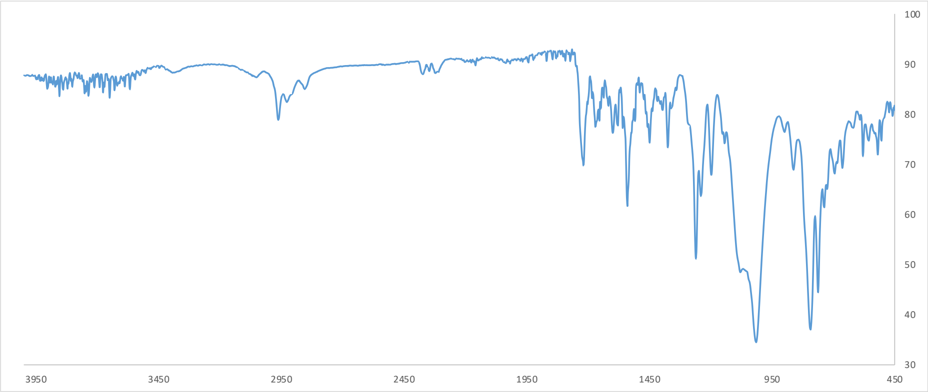

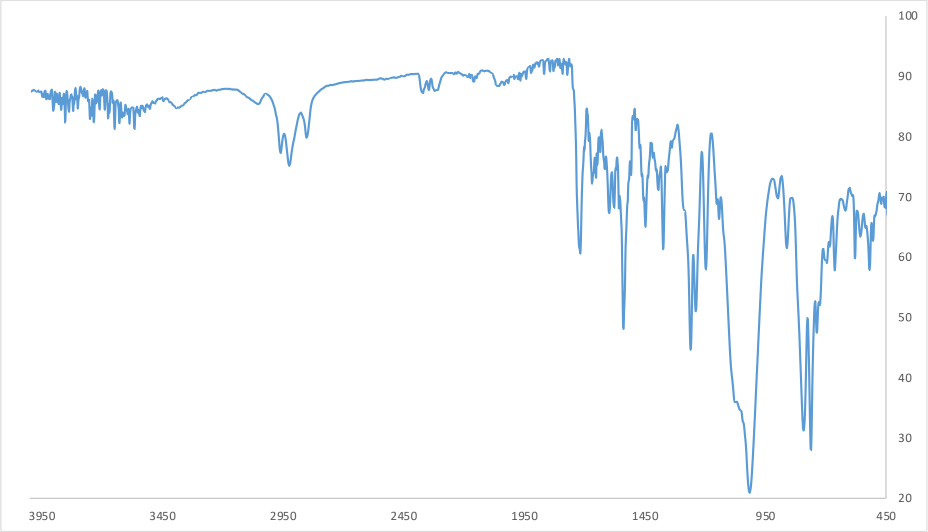

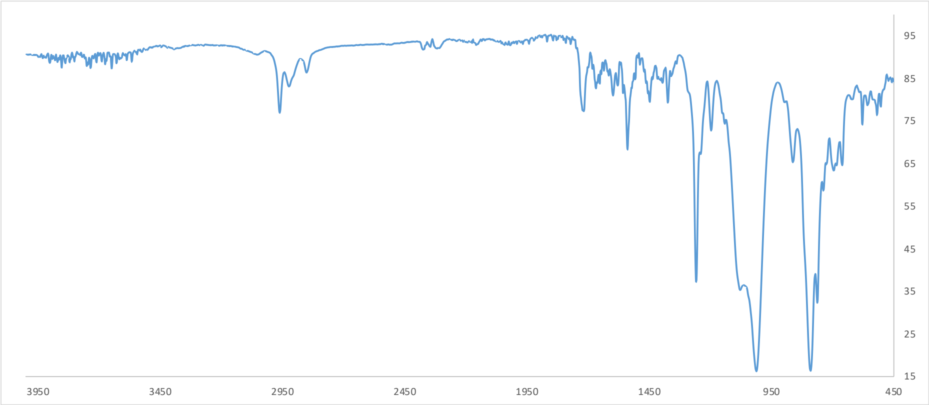

Figure S23. FT-IR spectra (wavenumber cm^{-1} *vs* transmission %) for the complexes, [Ir(epqc)($L^{1\text{-}3}$)]BF_4 (top to bottom).

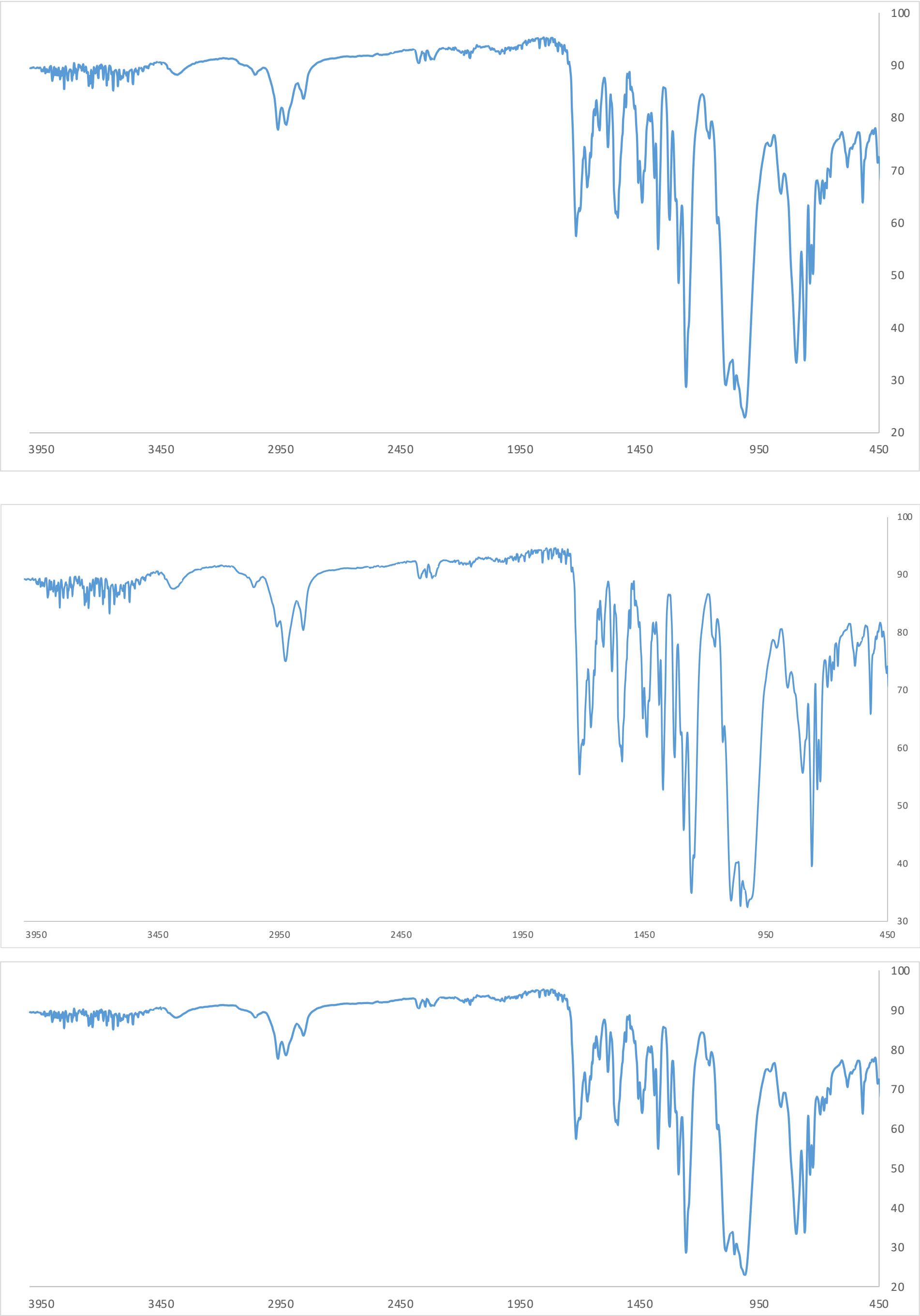

Figure S24. FT-IR spectra (wavenumber cm^{-1} *vs* transmission %) for the complexes, $[Ir(emptz)(L^{1-3})]BF_4$ (top to bottom).

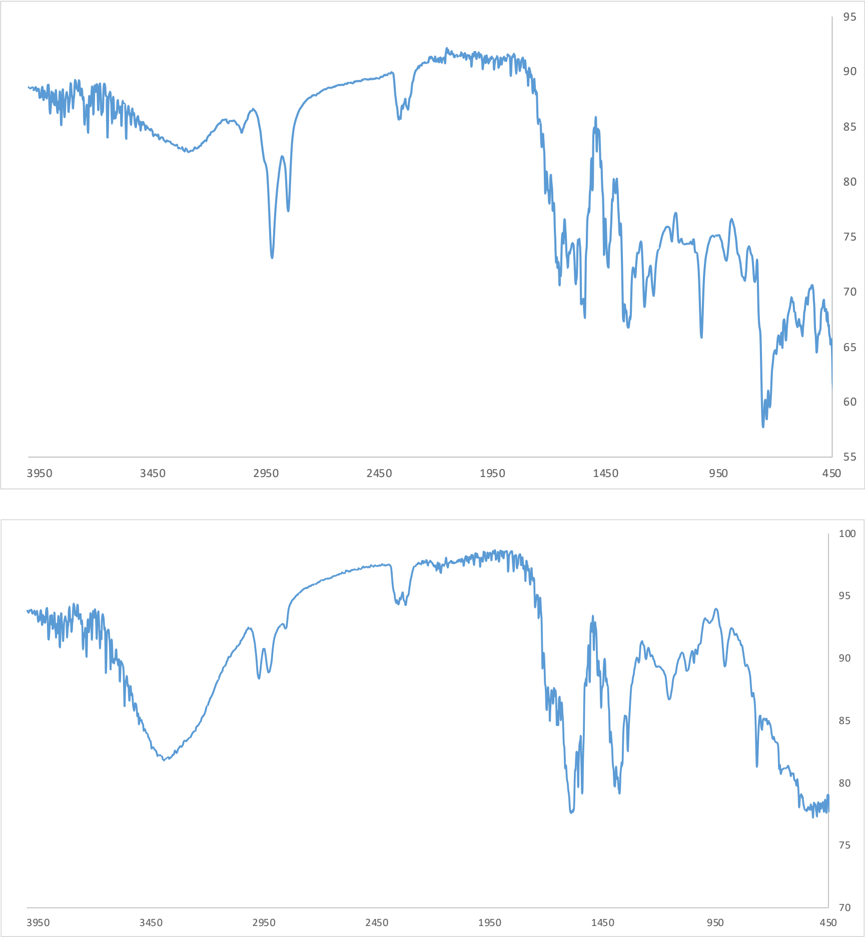

Figure S25. FT-IR spectra (wavenumber cm^{-1} *vs* transmission %) for [Ir(pqca)(L^3)]Cl (top) and [Ir(mptca)(L^3)]Cl (bottom).

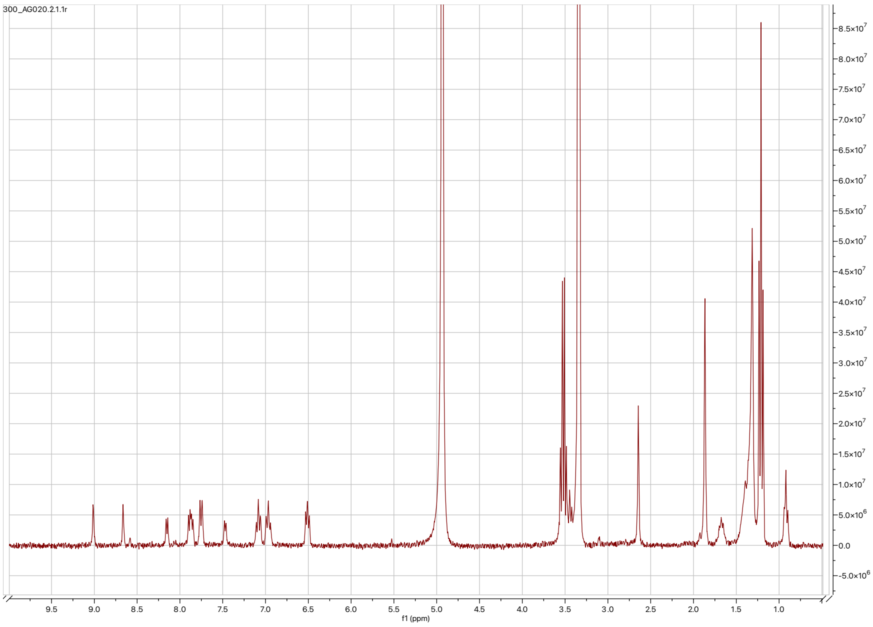

Figure S26. ^{1}H NMR spectrum of [Ir(mptca)$_2$(L^3)]Cl (in CD_3OD).

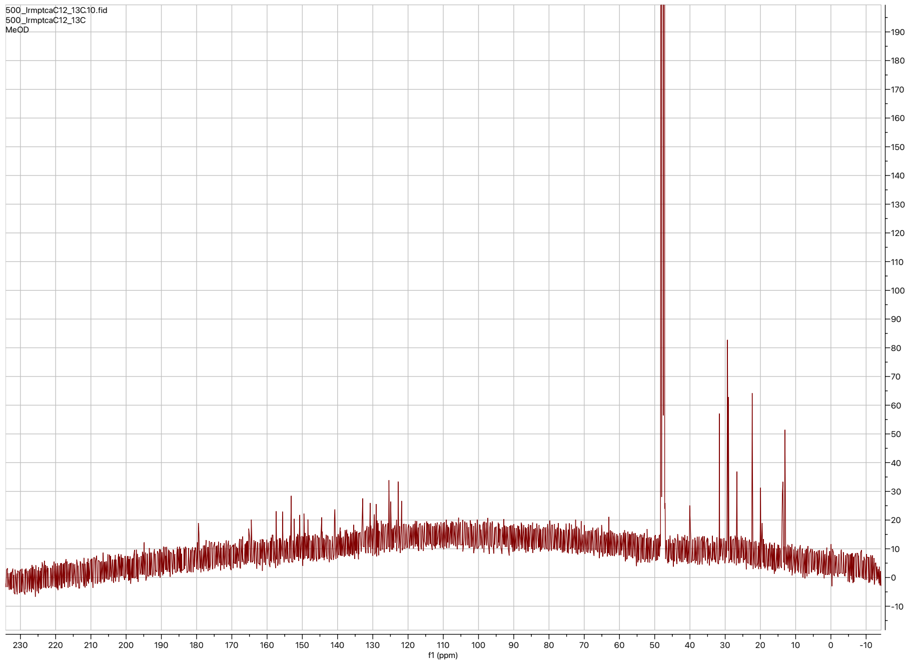

Figure S27. $^{13}C\{^{1}H\}$ NMR spectrum of $[Ir(mptca)_2(L^3)]Cl$ (in CD_3OD).

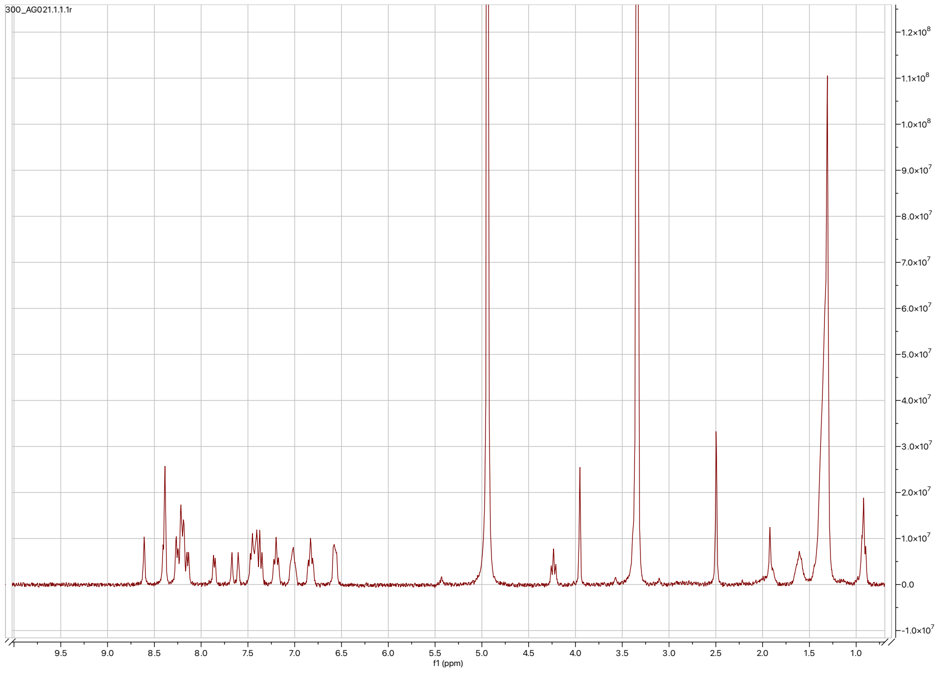

Figure S28. ^{1}H NMR spectrum of $[Ir(pqca)_2(L^3)]Cl$ (in CD_3OD).

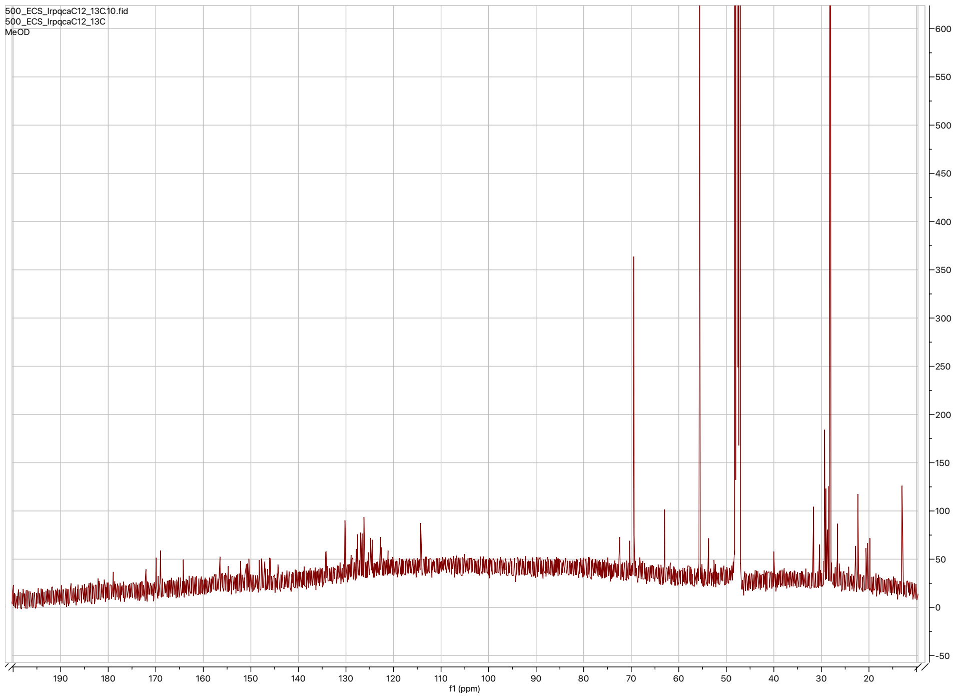

Figure S29. $^{13}C\{^1H\}$ NMR spectrum of $[Ir(pqca)_2(L^3)]Cl$ (in CD_3OD).

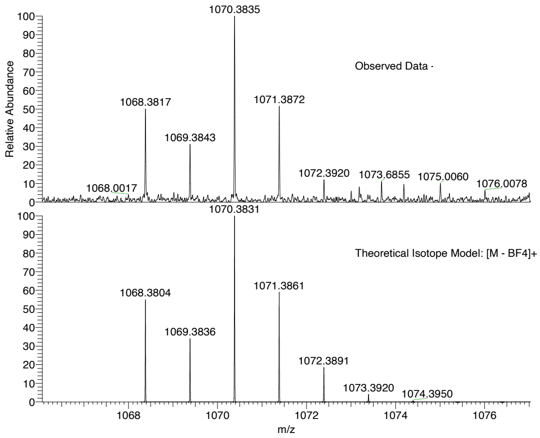

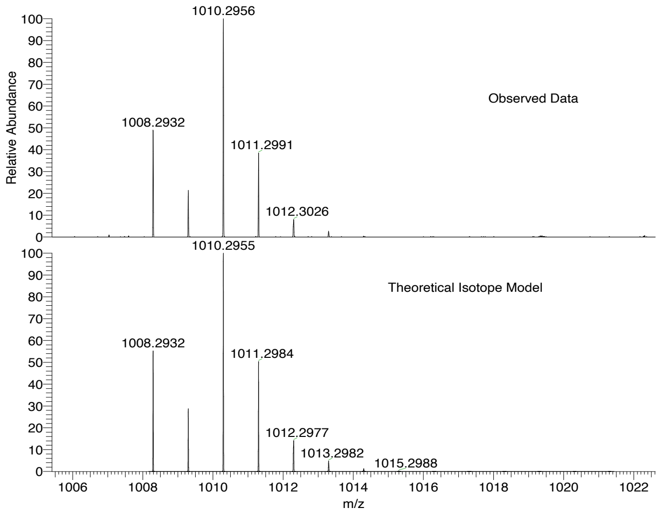

Figure S30. HRMS data for [Ir(pqca)$_2$(L^3)]Cl (top) and [Ir(mptca)$_2$(L^3)]Cl (bottom).

Table S1. Data collection parameters for the X-ray crystal structures.

Crystal	cis-$[Ir(epqc)_2(MeCN)_2]BF_4$	$[Ir(epqc)_2(L^3)]BF_4$
Formula	$C_{44}H_{44}BF_4IrN_4O_5$	$C_{62}H_{68}BF_4IrN_5O_{5.5}$
$D_{calc.}$/ g cm^{-3}	1.599	1.473
μ/mm^{-1}	3.322	2.437
Formula Weight	987.84	1250.22
Colour	red	red
Shape	needle-shaped	block-shaped
Size/mm^3	0.160×0.010×0.010	0.140×0.100×0.010
T/K	100(2)	100(2)
Crystal System	triclinic	triclinic
Space Group	*P*-1	*P*-1
a/Å	8.7992(2)	9.7337(2)
b/Å	16.6146(4)	16.3240(3)
c/Å	16.7086(4)	18.6845(4)
α/°	119.195(2)	100.368(2)
β/°	91.698(2)	91.400(2)
γ/°	102.782(2)	104.629(2)
V/Å^3	2052.24(9)	2818.06(10)
Z	2	2
Z'	1	1
Wavelength/Å	0.71075	0.71075
Radiation type	Mo K_α	Mo K_α
Θ_{min}/°	2.405	2.363
Θ_{max}/°	27.484	27.485
Measured Refl's.	40539	48362
Indep't Refl's	9389	12877
Refl's I≥2 σ(I)	8509	11538
R_{int}	0.0351	0.0285
Parameters	632	994
Restraints	415	603
Largest Peak	1.820	1.933
Deepest Hole	-1.049	-1.015
GooF	1.073	1.045
wR_2 (all data)	0.0674	0.0781
wR_2	0.0658	0.0758
R_1 (all data)	0.0312	0.0372
R_1	0.0264	0.0310

Table S2. Bond lengths and bond angles for the X-ray structures.

cis-$[Ir(epqc)_2(MeCN)_2]BF_4$		$[Ir(epqc)_2(L^3)]BF_4$	
Bond lengths (Å)			
Ir1–C1	1.991(3)	Ir1–C1	1.942(13)
Ir1–C21	1.997(3)	Ir1–C21	2.005(3)
Ir1–N21	2.089(2)	Ir1–C1B	2.11(3)
Ir1–N1	2.092(2)	Ir1–N1	2.082(8)
Ir1–N41	2.140(2)	Ir1–N1B	2.10(2)
Ir1–N51	2.150(2)	Ir1–N21	2.097(3)
		Ir1–N42	2.163(2)
		Ir1–N41	2.166(3)
Bond Angles (º)			
C1–Ir1–C21	89.24(11)	C1–Ir1–C21	88.9(9)
C1–Ir1–N21	93.57(10)	C21–Ir1–C1B	91.9(16)
C21–Ir1–N21	80.28(10)	C1–Ir1–N1	81.0(4)
C1–Ir1–N1	80.12(10)	C21–Ir1–N1	93.1(4)
C21–Ir1–N1	92.38(10)	C21–Ir1–N1B	92.6(9)
N21–Ir1–N1	170.43(9)	C1B–Ir1–N1B	77.2(7)
C1–Ir1–N41	175.99(10)	C1–Ir1–N21	96.5(3)
C21–Ir1–N41	92.70(10)	C21–Ir1–N21	80.17(12)
N21–Ir1–N41	83.31(9)	C1B–Ir1–N21	91.9(6)
N1–Ir1–N41	103.29(9)	N1–Ir1–N21	172.9(4)
C1–Ir1–N51	93.83(10)	N1B–Ir1–N21	166.8(7)
C21–Ir1–N51	174.44(9)	C1–Ir1–N42	99.0(9)
N21–Ir1–N51	104.13(9)	C21–Ir1–N42	170.96(11)
N1–Ir1–N51	83.60(9)	C1B–Ir1–N42	96.4(16)
N41–Ir1–N51	84.53(9)	N1–Ir1–N42	84.0(4)
		N1B–Ir1–N42	85.8(9)
		N21–Ir1–N42	103.01(9)
		C1–Ir1–N41	172.5(8)
		C21–Ir1–N41	97.12(12)
		C1B–Ir1–N41	166.8(12)
		N1–Ir1–N41	103.0(3)
		N1B–Ir1–N41	111.9(5)
		N21–Ir1–N41	80.19(10)
		N42–Ir1–N41	75.31(9)

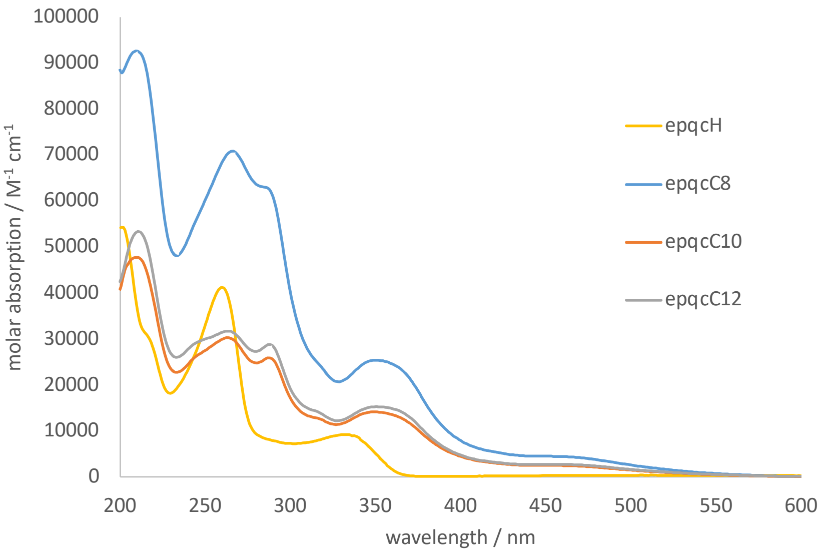

Figure S31. A comparison of the absorption spectra for free epqcH and $[Ir(epqc)_2(L^{1-3})]BF_4$ complexes.

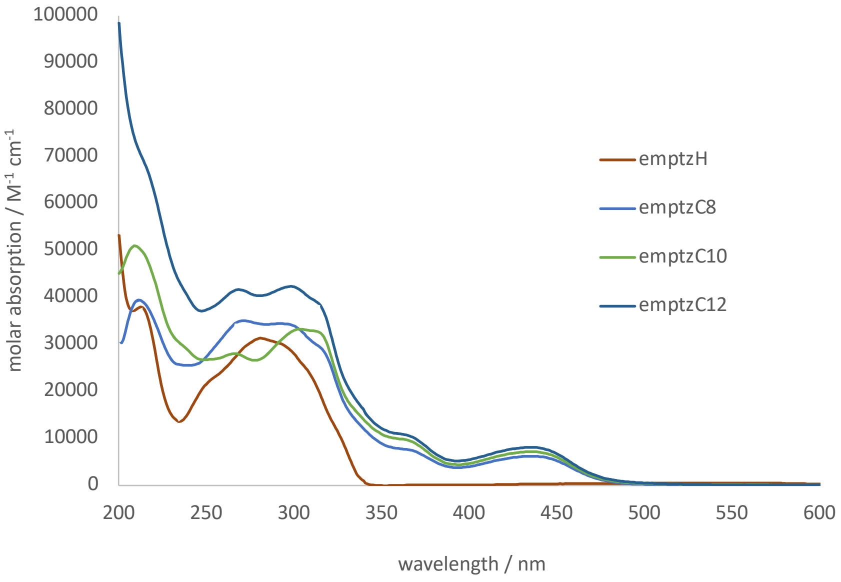

Figure S32. A comparison of the absorption spectra for free emptzH and $[Ir(emptz)_2(L^{1-3})]BF_4$ complexes.

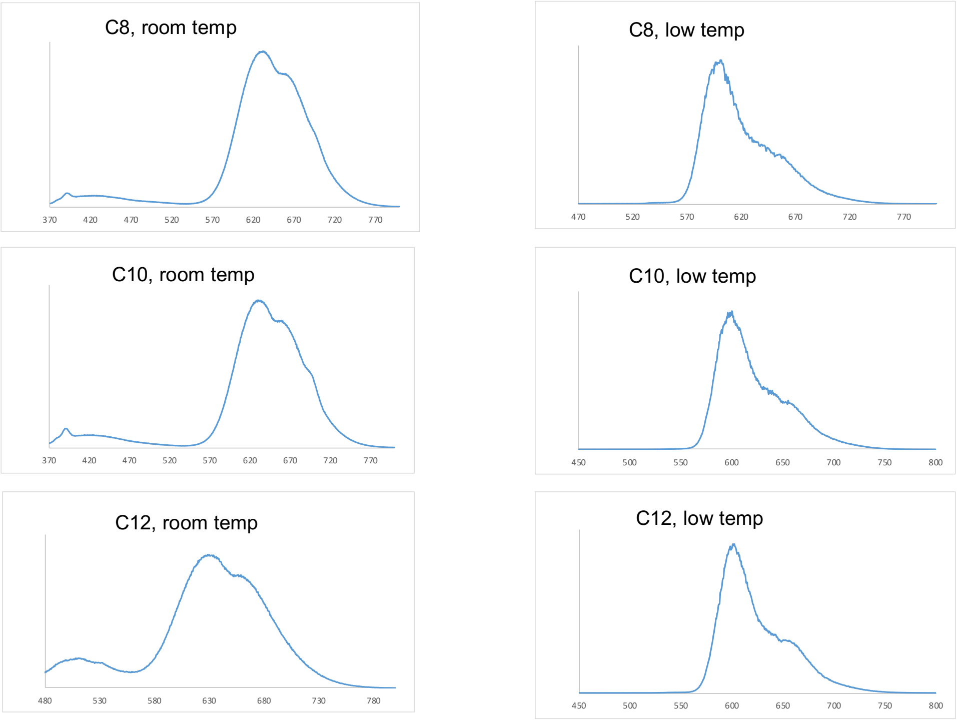

Figure S33. A comparison of the room and low temperature emission spectra for $[Ir(epqc)_2(L^{1\text{-}3})]BF_4$ complexes.

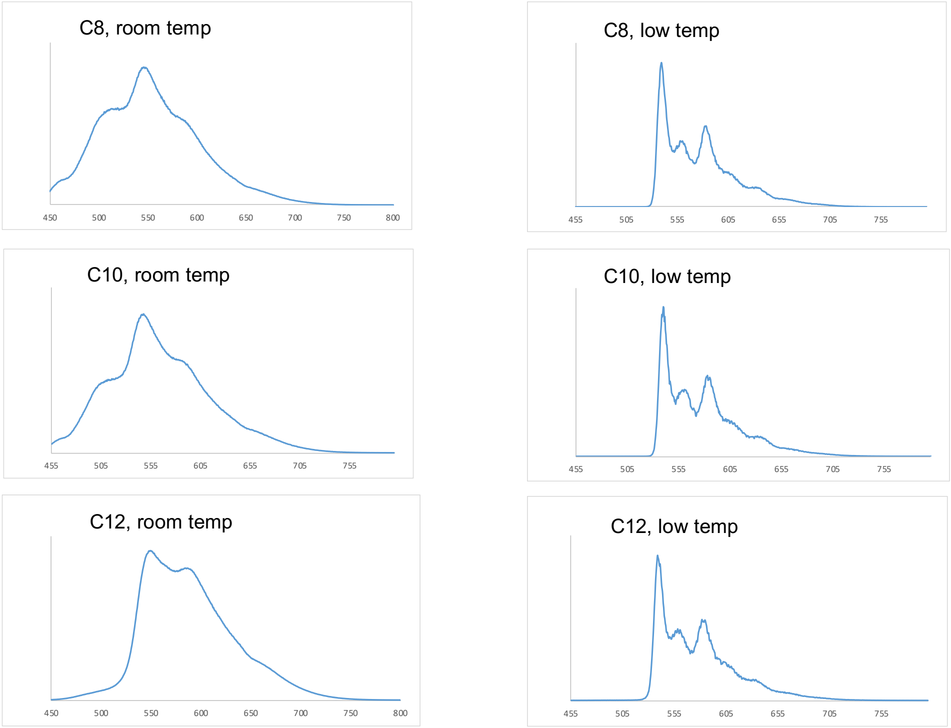

Figure S34. A comparison of the room and low temperature emission spectra for $[Ir(emptz)_2(L^{1\text{-}3})]BF_4$ complexes.
